# Lack of evidence for GWAS signals of exfoliation glaucoma working via monogenic loss-of-function mutation in the nearest gene

**DOI:** 10.1093/hmg/ddae088

**Published:** 2024-05-20

**Authors:** Kacie J Meyer, John H Fingert, Michael G Anderson

**Affiliations:** Department of Molecular Physiology and Biophysics, University of Iowa, 51 Newton Rd, Iowa City, IA 52242, United States; Institute for Vision Research, University of Iowa, 375 Newton Rd, Iowa City, IA 52242, United States; Institute for Vision Research, University of Iowa, 375 Newton Rd, Iowa City, IA 52242, United States; Department of Ophthalmology and Visual Sciences, University of Iowa, 200 Hawkins Dr, Iowa City, IA 52242, United States; Department of Molecular Physiology and Biophysics, University of Iowa, 51 Newton Rd, Iowa City, IA 52242, United States; Institute for Vision Research, University of Iowa, 375 Newton Rd, Iowa City, IA 52242, United States; Department of Ophthalmology and Visual Sciences, University of Iowa, 200 Hawkins Dr, Iowa City, IA 52242, United States; Center for the Prevention and Treatment of Visual Loss, Iowa City VA Health Care System, 601 Hwy 6 W, Iowa City, IA 52246, United States

**Keywords:** mouse model, exfoliation syndrome, GWAS, ocular, fibrillar exfoliative material

## Abstract

Purpose: Exfoliation syndrome (XFS) is a systemic disease of elastin-rich tissues involving a deposition of fibrillar exfoliative material (XFM) in the anterior chamber of the eye, which can promote glaucoma. The purpose of this study was to create mice with CRISPR/Cas9-induced variations in candidate genes identified from human genome-wide association studies (GWAS) and screen them for indices of XFS. Methods: Variants predicted to be deleterious were sought in the *Agpat1*, *Cacna1a*, *Loxl1*, *Pomp*, *Rbms3*, *Sema6a,* and *Tlcd5* genes of C57BL/6J mice using CRISPR/Cas9-based gene editing. Strains were phenotyped by slit-lamp, SD-OCT imaging, and fundus exams at 1–5 mos of age. Smaller cohorts of 12-mos-old mice were also studied. Results: Deleterious variants were identified in six targets; *Pomp* was recalcitrant to targeting. Multiple alleles of some targets were isolated, yielding 12 strains. Across all genotypes and ages, 277 mice were assessed by 902 slit-lamp exams, 928 SD-OCT exams, and 358 fundus exams. Homozygosity for *Agpat1* or *Cacna1a* mutations led to early lethality; homozygosity for *Loxl1* mutations led to pelvic organ prolapse, preventing aging. *Loxl1* homozygotes exhibited a conjunctival phenotype of potential relevance to XFS. Multiple other genotype-specific phenotypes were variously identified. XFM was not observed in any mice. Conclusions: This study did not detect XFM in any of the strains. This may have been due to species-specific differences, background dependence, or insufficient aging. Alternatively, it is possible that the current candidates, selected based on proximity to GWAS signals, are not effectors acting via monogenic loss-of-function mechanisms.

## Introduction

Exfoliation syndrome (XFS) is a systemic disease of elastin-rich tissues characterized by the production and accumulation of exfoliative material (XFM). Clinically, XFS is most recognized for its effect on the anterior chamber of the eye, where it is typically observed by slit-lamp exam as a white fibrillar deposit on the anterior surface of the lens, pupillary border of the iris, and other structures [1]. XFM also accumulates in the trabecular meshwork, where it is associated with multiple abnormalities that are thought to impede aqueous humor outflow, promoting increased intraocular pressure (IOP) and exfoliative glaucoma (XFG) [2, 3]. XFS is considered the leading cause of secondary glaucoma worldwide [4–6].

One of the resources needed to study mechanisms and potential treatments for XFS is an animal model [7–12]. Unfortunately, there are currently no widely accepted animal models of XFS that robustly resemble the human disease. One possibility for developing such a model comes from genetic studies. In recent years, large genome-wide association studies (GWAS) have identified multiple loci associated with XFS disease risk [13–16]. By manipulating the orthologs of candidates from these loci in mice, it should, in theory, be possible to derive a mouse model that is also prone to XFS. Here, we sought to develop animal models of XFS by performing a screen using CRISPR/Cas9 technology with inbred C57BL/6J mice. We induced variations predicted to be deleterious in genes previously associated with XFS, and phenotyped the resultant strains by slit-lamp and SD-OCT examinations. Our slit-lamp data indicate that individual variants in the *Agpat1*, *Cacna1a*, *Loxl1*, *Rbms3*, *Sema6a,* and *Tlcd5* genes do not result in detectable XFM accumulation in the anterior segment. A conjunctival phenotype, potentially of relevance to XFS, was discovered in mice homozygous for *Loxl1* mutation and a variety of other ocular genotype-specific defects with unknown relevance to XFS were also identified in some strains. We discuss these findings in terms of the challenges of modeling common multigenic disease with mice, and the complexity of GWAS loci themselves.

## Results

### Generation of CRISPR/Cas9-mediated variations

CRISPR/Cas9-based gene editing with C57BL/6J mice led to successful targeting of 6 of 7 targets. One gene (*Pomp*) was recalcitrant to targeting and two were especially prone to targeting, leading to multiple alleles for some candidates (including 5 alleles for *Loxl1* and 3 for *Sema6a*). Of the variants selected for carrying forward (Table 1), 6 were simple indels predicted to cause frameshifts and premature stop codons in the targeted exon (*Cacna1a^em1Andm^*, *Loxl1^em1Andm^*, *Loxl1^em2Andm^*, *Loxl1^em3Andm^*, *Sema6a^em1Andm^*, *Sema6a^em2Andm^*), 3 were compound events involving multiple deletion/insertion events in a single exon of a single candidate predicted to cause frameshifts and premature stop codons in the targeted exon (*Loxl1^em4Andm^*, *Loxl1^em5Andm^*, *Tlcd5^em1Andm^*), 2 were deletions encompassing both splice acceptor/donor sites and coding bases of the targeted exon (*Agpat1^em1Andm^*, *Rbms3^em1Andm^*), and 1 was a small deletion predicted to cause an in frame deletion of 4 amino acids (*Sema6a^em3Andm^*).

**Table 1:** Mutation summary.

**Gene**	**Summary**	**Official nomenclature**	**Allele Consequence**
*Agpat1*	175-bp del	*Agpat1^em1Andm^*	175-bp deletion encompassing 116 bp of coding sequence, splice donor of the targeted exon, and 59 bp of intron. Predicted to disrupt splicing.
*Cacna1a*	1-bp ins	*Cacna1a^em1Andm^*	1-bp coding sequence insertion in the targeted exon. Predicted to cause frameshift and premature STOP in the targeted exon.
*Loxl1*	41-bp del	*Loxl1^em1Andm^*	41-bp coding sequence deletion in the targeted exon. Predicted to cause frameshift and premature STOP in the targeted exon.
	58-bp del	*Loxl1^em2Andm^*	58-bp coding sequence deletion in the targeted exon. Predicted to cause frameshift and premature STOP in the targeted exon.
	2-bp ins	*Loxl1^em3Andm^*	2-bp coding sequence insertion in the targeted exon. Predicted to cause frameshift and premature STOP in the targeted exon.
	27-bp del + 1-bp del	*Loxl1^em4Andm^*	27-bp coding sequence deletion and a separate 1-bp coding sequence insertion, both in the targeted exon. Predicted to cause frameshift and premature STOP in the targeted exon.
	39-bp del + 8-bp del	*Loxl1^em5Andm^*	39-bp coding sequence deletion and a separate 8-bp coding sequence insertion, both in the targeted exon. Predicted to cause frameshift and premature STOP in the targeted exon.
*Rbms3*	13-bp del	*Rbms3^em1Andm^*	13-bp deletion encompassing the splice acceptor and the first 8 coding bases of the targeted exon. Predicted to disrupt splicing.
*Sema6a*	1-bp ins	*Sema6a^em1Andm^*	1-bp coding sequence insertion in the targeted exon. Predicted to cause frameshift and premature STOP in the next downstream exon.
	20-bp del	*Sema6a^em2Andm^*	20-bp coding sequence deletion in the targeted exon. Predicted to cause frameshift and premature STOP in the next downstream exon.
	12-bp del	*Sema6a^em3Andm^*	12-bp coding sequence deletion in the targeted exon. Predicted to cause in-frame deletion of 4 amino acids in the targeted exon.
*Tlcd5*	1-bp ins + 11-bp del	*Tlcd5^em1Andm^*	1-bp coding sequence insertion and a separate 11-bp coding sequence deletion in the targeted exon. Predicted to cause frameshift and premature stop in the targeted exon.

del = deletion.

ins = insertion.

bp = base pairs.

### Phenotypic analysis

From the combined strains, one cohort of 224 mice was studied with comprehensive longitudinal imaging of the anterior (Table 2) and posterior (Table 3) eye at Early (~1–2 month) and Mid (~4–5 month) time points. An independent cohort of 53 mice was studied at a single Old (~12 month) time point for sampling the effects of more extensive aging. From the combined analyses of the 277 mice in these cohorts, no primary or secondary indices of XFS were detected.

**Table 2:** Anterior segment phenotypes.

				**Central corneal thickness (CCT) μm, mean ± SD, n = mice**		**Anterior chamber depth (ACD) μm, mean ± SD, n = mice**		**Slit-lamp**		**Anterior Eye Summary**
**Gene**	**Allele**	**Genotype**	**Health**	**Early**	**Mid**	**Early**	**Mid**	**Early**	**Mid**	
*Agpat1*	em1Andm	WT		97.8 ± 1.8 (n = 4)	101.2 ± 4.6 (n = 3)	353.0 ± 29.4 (n = 4)	414.1 ± 12.0 (n = 4)	normal	normal	No anterior phenotypes
*Agpat1*	em1Andm	HET		99.0 ± 3.0 (n = 5)	101.8 ± 7.8 (n = 4)	354.2 ± 20.9 (n = 6)	413.8 ± 13.6 (n = 6)	normal	normal	
*Agpat1*	em1Andm	MUT	lethal	ND	ND	ND	ND	ND	ND	
*Cacna1a*	em1Andm	WT		100.2 ± 2.5 (n = 5)	103.1 ± 2.1 (n = 5)	354.4 ± 6.9 (n = 6)	425.2 ± 9.8 (n = 6)	normal	normal	Limited sample size: homozygotes have decreased CCT and decreased ACD
*Cacna1a*	em1Andm	HET		100.7 ± 2.8 (n = 8)	101.9 ± 4.5 (n = 7)	350.2 ± 20.5 (n = 8)	419.1 ± 15.5 (n = 8)	normal	normal	
*Cacna1a*	em1Andm	MUT	lethal	79.0 ± 3.5 (n = 2)^a^	ND	208.5 ± 5.7 (n = 2)^a^	ND	normal	ND	
*Loxl1*	All	WT		100.0 ± 3.4 (n = 13)	101.2 ± 3.0 (n = 11)	367.3 ± 16.8 (n = 13)	419.8 ± 7.6 (n = 11)	normal	normal	Robust context: homoygotes have decreased CCT, increased ACD, and a conjunctiva abnormality
*Loxl1*	All	HET		97.0 ± 4.4 (n = 28)	97.7 ± 3.0 (n = 30)	363.5 ± 22.0 (n = 31)	426.7 ± 14.6 (n = 30)	normal	normal	
*Loxl1*	All	MUT	POP	**90.5 ± 4.1 (n = 36)**	**90.3 ± 4.2 (n = 32)^b^**	**380.9 ± 23.0 (n = 36)**	**449.2 ± 22.5 (n = 34)^b^**	abnormal conjuctiva	normal	
*Loxl1*	em1Andm	HET		97.4 ± 2.8 (n = 7)	95.2 ± 2.6 (n = 7)	370.8 ± 23.7 (n = 8)	427.1 ± 14.1 (n = 7)	normal	normal	
*Loxl1*	em1Andm	MUT	POP	**94.3 ± 2.0 (n = 11)**	**88.3 ± 3.7 (n = 6)^b^**	379.4 ± 19.1 (n = 12)	462.2 ± 25.8 (n = 7)^b^	abnormal conjuctiva	normal	
*Loxl1*	em2Andm	HET		97.4 ± 2.9 (n = 7)	98.9 ± 2.7 (n = 7)	357.4 ± 23.5 (n = 7)	426.9 ± 10.2 (n = 7)	normal	normal	
*Loxl1*	em2Andm	MUT	POP	**87.5 ± 3.2 (n = 7)**	**89.2 ± 4.6 (n = 10)^b^**	**384.4 ± 9.5 (n = 7)**	442.9 ± 19.7 (n = 11)^b^	abnormal conjuctiva	normal	
*Loxl1*	em3Andm	HET		96.5 ± 4.8 (n = 3)	100.4 ± 3.3 (n = 4)	348.1 ± 5.6 (n = 4)	416.1 ± 17.1 (n = 4)	normal	normal	
*Loxl1*	em3Andm	MUT	POP	89.6 ± 3.4 (n = 9)	**93.9 ± 3.9 (n = 9)c**	**385.3 ± 17.0 (n = 9)**	456.0 ± 17.0 (n = 9)^b^	abnormal conjuctiva	normal	
*Loxl1*	em4Andm	HET		98.1 ± 3.3 (n = 4)	98.1 ± 1.9 (n = 5)	366.1 ± 15.3 (n = 5)	428.6 ± 6.5 (n = 5)	normal	normal	
*Loxl1*	em4Andm	MUT	POP	91.3 ± 0.4 (n = 2)	ND	385.8 ± 17.3 (n = 2)	ND	abnormal conjuctiva	normal	
*Loxl1*	em5Andm	HET		95.9 ± 7.4 (n = 7)	97.4 ± 2.9 (n = 7)	368.2 ± 27.1 (n = 7)	430.6 ± 21.5 (n = 7)	normal	normal	
*Loxl1*	em5Andm	MUT	POP	**88.6 ± 4.8 (n = 7)**	**88.8 ± 0.9 (n = 7)^b^**	371.8 ± 45.7 (n = 6)	436.6 ± 24.2 (n = 7)^b^	abnormal conjuctiva	normal	
*Rbms3*	em1Andm	WT		101.3 ± 4.9 (n = 8)	101.6 ± 6.0 (n = 7)	342.8 ± 20.8 (n = 6)	403.8 ± 20.2 (n = 6)	normal	normal	No anterior phenotypes
*Rbms3*	em1Andm	HET		103.7 ± 4.1 (n = 5)	99.3 ± 4.7 (n = 4)	351.9 ± 16.3 (n = 6)	413.8 ± 1.5 (n = 6)	normal	normal	
*Rbms3*	em1Andm	MUT		103.2 ± 4.0 (n = 5)	100.5 ± 8.4 (n = 6)	331.4 ± 18.2 (n = 6)	400.7 ± 10.9 (n = 7)	normal	normal	
*Sema6a*	All	WT		99.2 ± 3.9 (n = 8)	104.5 ± 2.2 (n = 8)	363.1 ± 19.0 (n = 10)	414.6 ± 14.1 (n = 9)	normal	normal	Limited context: homozygotes have decreased ACD
*Sema6a*	All null	HET		100.8 ± 4.2 (n = 14)	101.0 ± 4.6 (n = 14)	346.2 ± 20.3 (n = 16)	402.6 ± 13.2 (n = 16)	normal	normal	
*Sema6a*	All null	MUT		99.7 ± 6.4 (n = 8)	102.5 ± 4.1 (n = 12)	**340.2 ± 21.2 (n = 10)**	**395.8 ± 16.7 (n = 13)**	normal	normal	
*Sema6a*	em1Andm	HET		99.9 ± 2.4 (n = 8)	103.6 ± 3.4 (n = 7)	345.1 ± 21.6 (n = 8)	400.1 ± 12.0 (n = 8)	normal	normal	
*Sema6a*	em1Andm	MUT		100.3 ± 7.6 (n = 4)	103.3 ± 4.1 (n = 6)	336.9 ± 24.7 (n = 5)	389.6 ± 11.3 (n = 7)	normal	normal	
*Sema6a*	em2Andm	HET		101.9 ± 5.8 (n = 6)	98.4 ± 4.2 (n = 7)	347.3 ± 20.4 (n = 8)	405.1 ± 14.7 (n = 8)	normal	normal	
*Sema6a*	em2Andm	MUT		99.1 ± 6.1 (n = 4)	101.7 ± 4.3 (n = 6)	343.4 ± 19.2 (n = 5)	403.0 ± 20.1 (n = 6)	normal	normal	
*Sema6a*	em3Andm	HET		101.0 ± 2.7 (n = 6)	104.8 ± 2.7 (n = 6)	365.2 ± 5.6 (n = 5)	433.3 ± 13.2 (n = 5)	normal	normal	
*Sema6a*	em3Andm	MUT		101.0 ± 5.5 (n = 5)	106.4 ± 6.5 (n = 4)	358.1 ± 13.9 (n = 5)	422.9 ± 8.2 (n = 5)	normal	normal	
*Tlcd5*	em1Andm	WT		102.0 ± 4.8 (n = 8)	104.8 ± 2.5 (n = 8)	358.3 ± 16.2 (n = 8)	425.9 ± 9.0 (n = 8)	normal	normal	No anterior phenotypes
*Tlcd5*	em1Andm	HET		100.6 ± 4.7 (n = 8)	103.0 ± 2.4 (n = 5)	348.8 ± 16.7 (n = 8)	418.4 ± 11.5 (n = 8)	normal	normal	
*Tlcd5*	em1Andm	MUT		102.6 ± 5.1 (n = 7)	105.8 ± 5.2 (n = 6)	355.3 ± 17.2 (n = 7)	427.1 ± 17.2 (n = 7)	normal	normal	

POP = pelvic organ prolapse.

null = em1 + em2.

a 3.5 weeks.

b 2–5 month.

ND = no data.

**BOLD** = *P* < 0.05 (two-tailed *t*-test or ANOVA).

**Table 3:** Posterior segment phenotypes.

				**Retinal Thickness (RT) μm, mean ± SD, n = mice**		**Ganglion Cell Complex Thickness (GCC) μm, mean ± SD, n = mice**		**Fundus**	**Posterior eye summary**
**Gene**	**Allele**	**Genotype**	**Health**	**Early**	**Mid**	**Early**	**Mid**	**Mid**	
*Agpat1*	em1Andm	WT		208.9 ± 3.4 (n = 4)	208.8 ± 2.7 (n = 4)	57.5 ± 1.2 (n = 4)	56.9 ± 1.6 (n = 4)	normal	No posterior phenotypes
*Agpat1*	em1Andm	HET		211.0 ± 3.8 (n = 6)	211.6 ± 4.0 (n = 6)	58.1 ± 1.8 (n = 6)	57.4 ± 2.3 (n = 6)	normal	
*Agpat1*	em1Andm	MUT	lethal	ND	ND	ND	ND	ND	
*Cacna1a*	em1Andm	WT		208.3 ± 3.2 (n = 6)	206.1 ± 1.5 (n = 6)	58.3 ± 0.8 (n = 6)	56.4 ± 1.9 (n = 6)	normal	Limited context: homozygotes have reduced retinal lamination
*Cacna1a*	em1Andm	HET		209.8 ± 2.9 (n = 8)	208.7 ± 3.7 (n = 8)	58.0 ± 1.5 (n = 8)	56.8 ± 2.1 (n = 8)	normal	
*Cacna1a*	em1Andm	MUT	lethal	203.3 ± 4.7 (n = 2)^a^	ND	58.3 ± 1.9 (n = 2)^a^	ND	ND	
*Loxl1*	All	WT		208.0 ± 2.8 (n = 13)	209.1 ± 2.3 (n = 11)	56.7 ± 1.3 (n = 13)	56.5 ± 1.6 (n = 11)	normal	Robust context: homozygotes have modestly decreased GCC, especially at early time point
*Loxl1*	All	HET		208.2 ± 2.7 (n = 31)	209.8 ± 2.7 (n = 30)	56.8 ± 1.6 (n = 31)	56.4 ± 1.3 (n = 30)	normal	
*Loxl1*	All	MUT	POP	207.6 ± 4.0 (n = 37)	208.5 ± 4.0 (n = 37)^b^	**55.5 ± 1.8 (n = 37)**	56.0 ± 2.4 (n = 37)^b^	normal	
*Loxl1*	em1Andm	HET		207.9 ± 2.6 (n = 8)	211.0 ± 3.3 (n = 7)	56.5 ± 1.8 (n = 8)	57.4 ± 1.0 (n = 7)	normal	
*Loxl1*	em1Andm	MUT	POP	209.5 ± 3.1 (n = 12)	209.0 ± 2.2 (n = 7)^b^	55.9 ± 1.8 (n = 12)	**55.2 ± 1.6 (n = 7)^b^**	normal	
*Loxl1*	em2Andm	HET		206.9 ± 1.8 (n = 7)	208.7 ± 3.1 (n = 7)	56.3 ± 1.4 (n = 7)	55.9 ± 1.4 (n = 7)	normal	
*Loxl1*	em2Andm	MUT	POP	204.3 ± 3.1 (n = 7)	207.6 ± 5.2 (n = 11)^b^	**54.0 ± 1.0 (n = 7)**	55.5 ± 2.0 (n = 11)^b^	normal	
*Loxl1*	em3Andm	HET		210.1 ± 3.4 (n = 4)	211.1 ± 1.5 (n = 4)	57.1 ± 1.9 (n = 4)	56.2 ± 1.0 (n = 4)	normal	
*Loxl1*	em3Andm	MUT	POP	209.7 ± 4.3 (n = 9)	210.4 ± 3.7 (n = 9)^b^	55.5 ± 1.6 (n = 9)	56.9 ± 2.9 (n = 9)^b^	normal	
*Loxl1*	em4Andm	HET		210.2 ± 2.2 (n = 5)	209.2 ± 1.1 (n = 5)	57.3 ± 1.6 (n = 5)	56.5 ± 1.3 (n = 5)	normal	
*Loxl1*	em4Andm	MUT	POP	202.9 ± 6.1 (n = 2)	ND	53.3 ± 2.1 (n = 2)	ND	ND	
*Loxl1*	em5Andm	HET		207.6 ± 3.1 (n = 7)	208.7 ± 2.5 (n = 7)	57.1 ± 1.6 (n = 7)	55.1 ± 1.2 (n = 7)	normal	
*Loxl1*	em5Andm	MUT	POP	206.9 ± 2.1 (n = 7)	208.1 ± 3.0 (n = 9)^b^	56.7 ± 1.2 (n = 7)	56.1 ± 2.9 (n = 9)^b^	normal	
*Rbms3*	em1Andm	WT		206.2 ± 4.6 (n = 9)	204.8 ± 2.4 (n = 9)	55.9 ± 2.4 (n = 9)	56.3 ± 1.4 (n = 9)	normal	Limited context: homozygotes have decreased RT at early time point
*Rbms3*	em1Andm	HET		209.8 ± 3.1 (n = 7)	207.2 ± 2.6 (n = 7)	57.5 ± 2.1 (n = 7)	57.4 ± 2.0 (n = 7)	normal	
*Rbms3*	em1Andm	MUT		**201.2 ± 3.7 (n = 7)**	204.2 ± 2.9 (n = 8)	54.8 ± 1.6 (n = 7)	56.2 ± 1.8 (n = 8)	normal	
*Sema6a*	All	WT		207.8 ± 3.6 (n = 13)	209.3 ± 3.3 (n = 13)	55.7 ± 1.7 (n = 13)	56.4 ± 2.5 (n = 13)	normal	Robust context: homozygotes have increased GCC with an atypical appearance
*Sema6a*	All null	HET		206.8 ± 3.3 (n = 16)	204.3 ± 4.0 (n = 16)	55.0 ± 1.7 (n = 16)	55.1 ± 2.3 (n = 16)	normal	
*Sema6a*	All null	MUT		**212.3 ± 3.4 (n = 10)**	209.1 ± 3.1 (n = 13)	**60.2 ± 1.5 (n = 10)**	**59.8 ± 1.8 (n = 13)**	normal	
*Sema6a*	em1Andm	HET		207.0 ± 3.6 (n = 8)	204.9 ± 1.8 (n = 8)	55.1 ± 1.7 (n = 8)	55.6 ± 1.4 (n = 8)	normal	
*Sema6a*	em1Andm	MUT		210.7 ± 4.3 (n = 5)	**209.1 ± 3.6 (n = 7)**	**59.8 ± 2.0 (n = 5)**	**60.0 ± 1.9 (n = 7)**	normal	
*Sema6a*	em2Andm	HET		206.6 ± 3.0 (n = 8)	203.6 ± 5.5 (n = 8)	54.8 ± 1.7 (n = 8)	54.6 ± 3.0 (n = 8)	normal	
*Sema6a*	em2Andm	MUT		**213.8 ± 1.4 (n = 5)**	**209.2 ± 2.8 (n = 6)**	**60.5 ± 1.1 (n = 5)**	**59.5 ± 1.7 (n = 6)**	normal	
*Sema6a*	em3Andm	HET		209.8 ± 2.6 (n = 6)	206.9 ± 1.9 (n = 6)	57.0 ± 0.9 (n = 6)	55.4 ± 0.9 (n = 6)	normal	
*Sema6a*	em3Andm	MUT		204.4 ± 5.2 (n = 7)	207.1 ± 1.9 (n = 7)	55.7 ± 2.1 (n = 7)	56.8 ± 0.8 (n = 7)	normal	
*Tlcd5*	em1Andm	WT		209.3 ± 1.7 (n = 8)	208.0 ± 3.0 (n = 8)	57.6 ± 0.9 (n = 8)	55.9 ± 2.6 (n = 8)	normal	No posterior phenotypes
*Tlcd5*	em1Andm	HET		207.4 ± 3.9 (n = 8)	206.2 ± 2.9 (n = 8)	56.0 ± 2.0 (n = 8)	55.1 ± 1.5 (n = 8)	normal	
*Tlcd5*	em1Andm	MUT		206.6 ± 3.8 (n = 7)	208.2 ± 1.7 (n = 7)	57.5 ± 2.0 (n = 7)	55.7 ± 1.9 (n = 7)	normal	

POP = pelvic organ prolapse.

null = em1 + em2.

a 3.5 weeks.

b 2–5 month.

ND = no data.

**BOLD** = *P* < 0.05 (two-tailed *t*-test or ANOVA).

**Figure 1:**
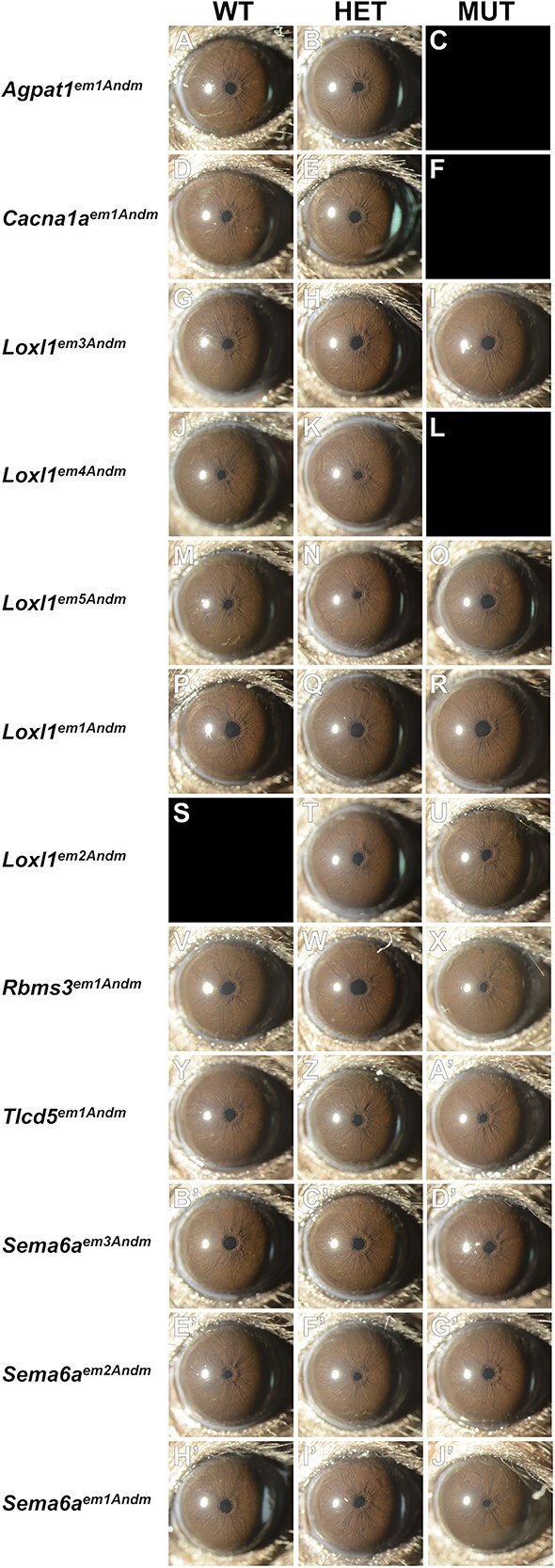
Representative images showing anterior segment slit-lamp images from mid time point mice. Empty fields indicate no data for a particular genotype. Note that no indices of XFS were present and eyes were typically healthy in appearance. All mice = 4–5 month.

A collective 902 slit-lamp exams (Complete image dataset available at doi: 10.25820/data.006799 [17]; complete data description in Supplemental File 1) detected no XFM accumulation, no XFS-associated iris transillumination defects, no pigment dispersion, and no pupillary ruff atrophy in any eyes (Mid time point, Fig. 1; Early time point Supplemental File 2). Lens opacities were observed, especially with advancing age (0/394 eyes Early, 5/394 eyes Mid, 20/98 eyes Old), but were not associated with any particular strain or genotype. Buphthalmia, which is sometimes associated with glaucomatous IOP in mice, did not occur.

**Figure 2:**
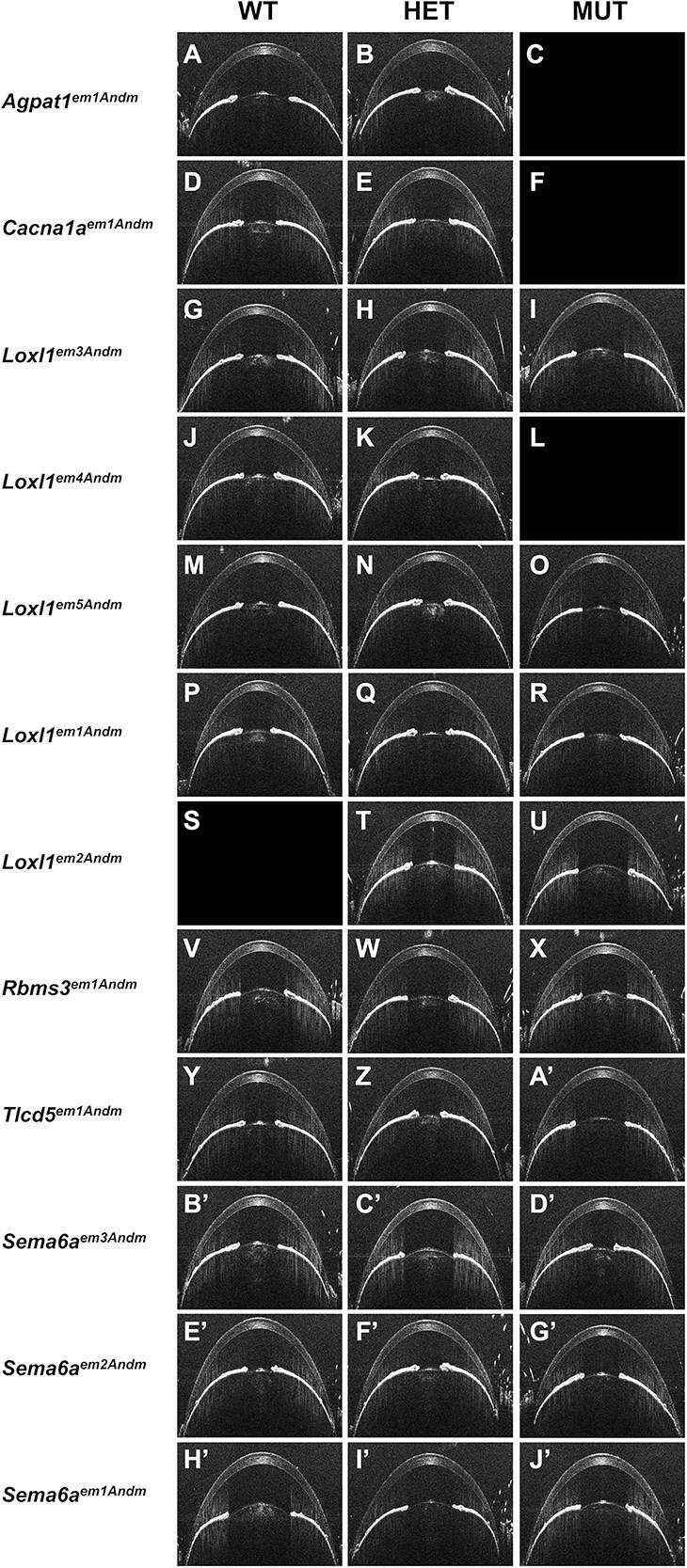
Representative anterior segment SD-OCT images from mid time point mice. Empty fields indicate no data for a particular genotype. Note that gross qualitative appearances are similar across genotypes. A recurrent modest increase in anterior chamber depth for *Loxl1* homozygotes (panels I, O, R, U) is apparent. All mice = 4–5 month.

**Figure 3:**
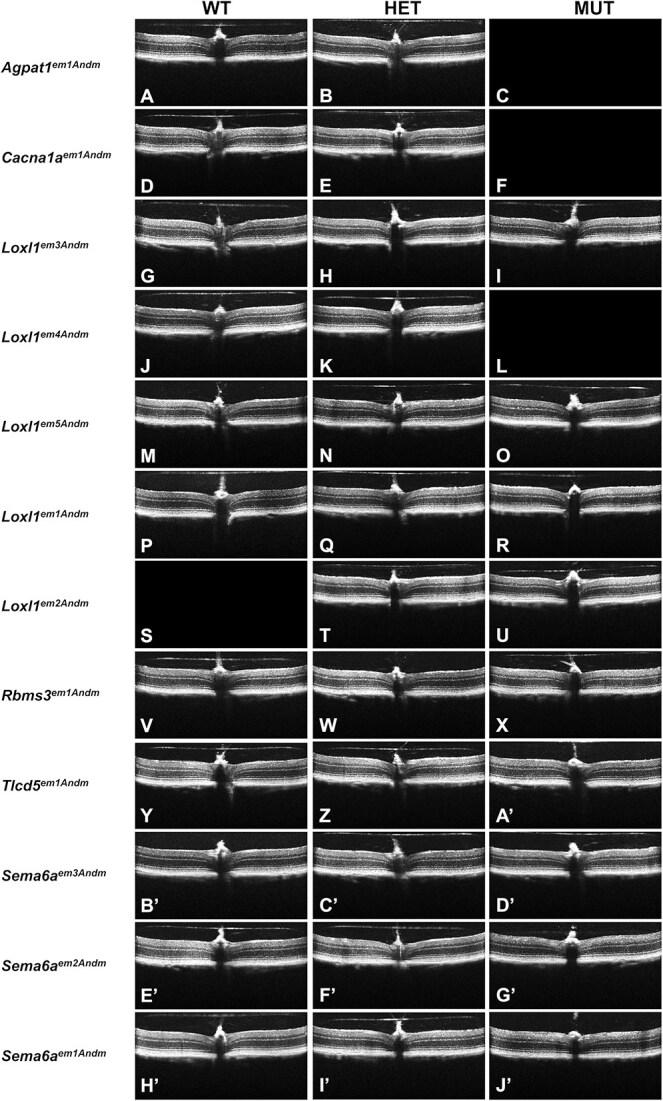
Representative retinal SD-OCT images from mid time point mice. Empty fields indicate no data for a particular genotype. Note that gross qualitative appearances are similar across genotypes. A recurrent modest change in appearance of the ganglion cell complex is apparent in *Sema6a^em2Andm^* (G’) and *Sema6a^em1Andm^* (J’). All mice = 4–5 month.

**Figure 4:**
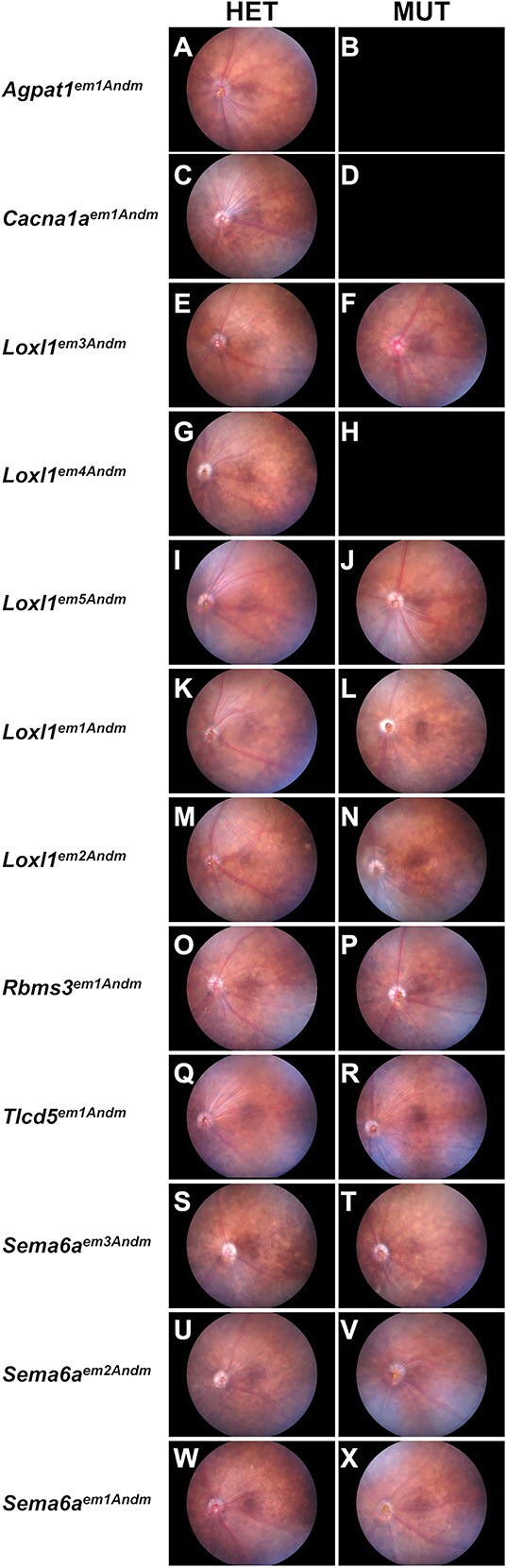
Representative images showing fundus images from mid time point mice. Empty fields indicate no data for a particular genotype. Note that gross qualitative appearances are similar across genotypes. All mice = 4–5 month.

A collective 928 SD-OCT dual exams of the anterior segment and retina (complete data description and statistical comparisons in Supplemental Files 3 and 4) detected largely healthy tissues. The anterior segment had a normal appearance at both the Early (Supplemental File 5) and the Mid (Fig. 2) time points. Similarly, the retina had a normal appearance at both the Early (Supplemental File 6) and the Mid (Fig. 3) time points. Finally, no reproducible qualitative strain- or genotype-specific abnormalities were detected in a collective 358 fundus exams at the Mid time point (Fig. 4).

While expected hallmarks of XFS or XFG were absent, multiple other genotype-specific phenotypes were variously identified, as described below:

#### Loxl1

**Figure 5:**
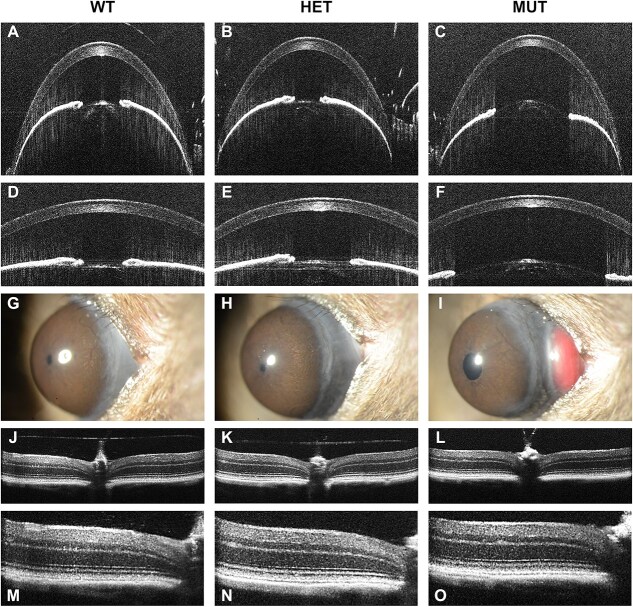
Phenotypes of *Loxl1* mutant mice. (A–F) Anterior chamber SD-OCT images from mid time point of mice from *Loxl1^em2Andm^*. On close examination, increased anterior chamber depth and decreased corneal thickness are present in the homozygote, amongst tissues that otherwise appear healthy. (G–I) Anterior segment slit-lamp images showing an example of orbital gland tissue that has temporarily slid anteriorly into the temporal bulbar conjunctiva at the lateral palpebral commissure (large red protuberance) in the homozygote. (J–O) Retinal SD-OCT images showing a qualitatively normal-appearing retina, despite modest quantitative differences that were measured.

From the 5 new *Loxl1*-mutant strains, multiple robust ocular phenotypes (Fig. 5) and one non-ocular phenotype were detected. Homozygotes from all 5 strains developed pelvic organ prolapse [18–20], as has been previously noted in other *Loxl1-*mutant strains [18–20]. Almost all homozygotes were euthanized shortly following the exams at 4 months due to the severity of pelvic organ prolapse. Consequently, the oldest homozygotes able to be examined were 181 and 190 days of age.

With anterior segment SD-OCT imaging, differences were detected for ACD in the binned comparisons at the Early (*P* = 0.0048) and Mid (*P* = 6.1 × 10^−7^) time points, with homozygous mutants from the *Loxl1* CRISPR/Cas9-induced mutant strains having a 1%–11% increase in ACD compared to other genotypes (Fig. 5A–C). Increased ACD was also statistically supported in 2 pairwise comparisons and apparent as a trend in all 7 of the remaining pairwise comparisons (Table 2). As expected based on findings of other studies [21], ACD exhibited a sex difference, with males having increased ACD compared to females (Supplemental File 3). Genotype of the *Loxl1* CRISPR/Cas9-induced mutant strains also significantly influenced CCT, as has been previously reported in studies of knock-out mice with a targeted *Loxl1* mutation [20]. In the binned comparisons, statistically significant changes in CCT were detected at the Early (*P* = 2.7 × 10^−11^) and Mid (*P* = 1.2 × 10^−14^) time points. Homozygous mutants were noted to have a ~3%–13% reduction in CCT compared to other genotypes (Fig. 5D–F). The decrease in total CCT involved reductions in both corneal stromal thickness and corneal epithelial thickness (Supplemental File 4). Decreased CCT was also statistically supported in 7 of 9 pairwise comparisons and apparent as a trend in 1 of 2 remaining pairwise comparisons (Table 2).

During slit-lamp exam, a conjunctival phenotype among *Loxl1* homozygotes was detected in which orbital gland tissue would temporarily slide anteriorly into the temporal bulbar conjunctiva at the lateral palpebral commissure (Fig. 5G–I). This phenomenon could sometimes be exaggerated by the way in which the mouse was held, becoming more likely to be observed with increasing proptosis of the eye. This phenotype was not recognized until midway through the project, so it was not initially documented. After its recognition, 23 of 25 young homozygous *Loxl1* mutants examined exhibited the phenotype in at least one eye—typically characterized by a large red protuberance. By comparison, only 5 of 47 Early mice of other genotypes assessed for the trait had it any form—typically a small red area that was not protuberant. Likewise, 29 of 30 Mid aged homozygous *Loxl1* mutants exhibited the phenotype, while only 14 of 77 mice of other genotypes had it any form. Only *Loxl1* homozygous mutants were observed to express the phenotype at more than one time point. Aside from the conjunctival phenotype, eyes appeared normal and healthy by slit-lamp exam.

With retinal SD-OCT imaging, homozygous mutants had a modest (~2%) decrease in GCC thickness with an otherwise healthy appearing retina (Fig. 5J–O). Decreased GCC thickness was statistically supported in the binned comparison at the Early time point (*P* = 0.0031) and as a trend at the Mid time point. Similarly, decreased GCC in *Loxl1* mutants was statistically supported in 2 pairwise comparisons of genotype within individual strains and was apparent as a trend in 5 of 9 remaining pairwise comparisons (Table 3). Finally, there was a corresponding genotype-specific modest reduction in overall retinal thickness (RT) from the binned comparisons, which was evident as a trend but not statistically significant.

#### Sema6a

**Figure 6:**
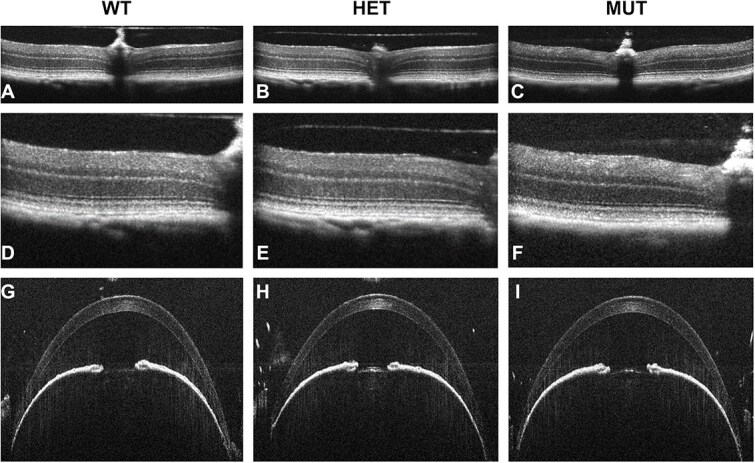
Phenotypes of *Sema6a* mutant mice. (A–F) Retinal SD-OCT images, shown at two magnifications from the same eyes, from *Sema6a^em2Andm^*. On close examination, the homozygote has a ganglion cell layer that appears “coarse” with variable hyper-reflectivity, as well as modestly thicker. (G–I) Anterior segment SD-OCT images showing a qualitatively normal-appearing anterior chamber, despite modest quantitative differences.

From the new *Sema6a*-mutant strains, a robust ocular phenotype was detected; the strains otherwise appeared overtly healthy. While slit-lamp and fundus exams detected no genotype-specific abnormalities, SD-OCT imaging detected an abnormal appearance of the GCC in homozygous mutants from 2 of the strains (*Sema6a^em1Andm^* and *Sema6a^em2Andm^*). In these mice the GCC appeared “coarse” with variable hyper-reflectivity within the layer (Fig. 6A–C). Differences were detected for GCC thickness in the binned comparisons at the Early (*P* = 6.1 × 10^−9^) and Mid (*P* = 4.6 × 10^−6^) time points in which homozygous mutants had an ~6%–9% increase in GCC compared to other genotypes. Increased GCC was also statistically supported in 4 of 6 pairwise comparisons, all from the strains having a coarse GCC appearance (Table 3). There was a corresponding increase in RT in the binned comparisons at the Early time point (*P* = 0.0029; Fig. 6D–F). Increased RT was also statistically supported in 3 pairwise comparisons, all from the strains having a coarse GCC appearance. With anterior segment SD-OCT imaging, the binned comparison differences detected differences for ACD at the Early (*P* = 0.0426) and Mid (*P* = 0.0208) time points, with homozygous mutants from the “coarse GCC” strains having an ~2%–6% reduction in ACD compared to other genotypes (Fig. 6G–I). Decreased ACD was not statistically supported in any of the individual pairwise comparisons (Table 2).

#### Cacna1a

From the new *Cacna1a*-mutant strain, multiple ocular phenotypes and a severe non-ocular phenotype were observed. Matching the findings from studies of other *Cacna1a* mutant strains [22, 23], homozygosity for the *Cacna1a^em1Andm^* mutation led to pups with an overtly wobbly gait. From initial crosses, four homozygotes were raised, without weaning, to 24 days of age before being euthanized. No ocular abnormalities were detected by slit-lamp exams of two homozygotes at 24 days of age. Both mice appeared to tolerate the exam well, but died before SD-OCT exams could be conducted the next day. The final two homozygotes were examined only by SD-OCT at 24 days of age, showing reduced retinal lamination, small eyes, shallow anterior chambers, and open pupils (Supplemental File 5F). Crosses were subsequently modified to prevent further potential births of homozygotes. No ocular phenotypes were detected in heterozygotes.

#### Agpat1

Matching the findings from studies of other *Agpat1* mutant strains [24], homozygosity for *Agpat1^em1Andm^* mutation led to a visibly smaller body size and lethality prior to weaning. No homozygotes survived long enough for an ocular exam. No ocular phenotypes were detected in heterozygotes.

#### Rbms3

From the new *Rbms3*-mutant strain, one ocular phenotype was detected in a limited context; the strain otherwise appeared overtly healthy. In an ANOVA comparison, genotype had a statistically significant influence on RT in which homozygous mutants had an ~2%–4% decrease in RT at the Early time point compared to other genotypes (*P* = 0.0023; Table 3).

### Additional phenotypic observations

In addition to the genotype-specific phenotypes described above, sporadic phenotypes that occurred across multiple strains were also noted (Supplemental File 7). Gross ocular abnormalities were uncommon in this cohort, with micro- or anophthalmia observed in 2/392 eyes at the Early time point (Supplemental File 7A and 7B), and Peters anomaly observed in 7/392 eyes at the Early time point (Supplemental File 7C and 7D). Notably, there were no observations of bilateral occurrence, with a propensity for occurrence in the right eye (8/9 instances). Additional observations of more subtle unilateral phenotypes include small strands connecting bulbs of the pupillary ruff (“pupil loops”; Supplemental File 7E and 7F), avulsed retinal vessels (Supplemental File 7G and 7H), corneal deposits consistent with band keratopathy (Supplemental File 7I), retinal dysplasias (Supplemental File 7J), differences in iris appearance (Supplemental File 7K), corneal endothelium abnormalities (Supplemental File 7L), iridocorneal adhesions concurrent with retinal/optic nerve dysplasia (Supplemental File 7M and 7N, respectively), anterior vitreous opacities proximal to the posterior lens (Supplemental File 7O and 7P, vitreous pigment “clumps” that appear to be associated with the hyaloid vasculature (Supplemental File 7Q and 7R), and retinal pigmentation abnormalities ranging in severity from a few isolated spots to geographic atrophy (Supplemental File 7S and 7T, respectively).

## Discussion

The need for an animal model of XFS/XFG is broadly appreciated [7–12]. While this screen did not find the robust mouse model of XFS that was our motivation, there were other advances. A straight-forward hypothesis that monogenic loss-of-function mutations in these candidates might cause overt classical symptoms of XFS has been challenged. With negative data, it’s currently unknown whether this is the consequence of using the wrong species, strain, ages; a requirement for polygenic mutations; or some other variable. It is also possible that these genes identified as risk factors by GWAS have a smaller role in XFS pathogenesis then has been previously considered. Regardless, the data suggest that developing a mouse model of XFS that involves XFM accumulations will require different strategies.

There is some uncertainty in the mechanisms linking XFS to exfoliative glaucoma. However, a predominantly held hypothesis suggests abnormalities associated with XFM in the iridocorneal angle impede aqueous humor outflow, promoting increased IOP and exfoliative glaucoma [2, 3]. Several questions remain regarding the mechanisms leading to XFM formation, how genetic and environmental risk factors contribute to XFM formation, and what interventions might disrupt the formation of XFM or its contributions to elevated IOP. To assist in addressing these questions, the chief feature desired in a mouse model of XFS is a robust accumulation of XFM in the anterior chamber. In the screening from our current experiments, there were multiple opportunities for observing anterior chamber defects, had they existed. Our primary observations and photodocumentation thoroughly demonstrate the absence of XFM accumulation or other expectations for XFS. The most common site of XFM accumulation is on the anterior lens and pupillary border [1, 4, 5, 25, 26], which can be readily observed with the slit-lamp in mice [25, 27]. Unfortunately, we did not detect XFM in any of the 12 mutant mouse strains we examined. XFS is associated with characteristic changes to the iris pigment epithelium that result in a characteristic pattern of iris transillumination defects [1, 28], pupillary ruff atrophy [1, 26], and pigment dispersion [1, 29], all of which have been identified in other studies of mice [30–32], but were normal in our 12 new mouse lines. XFS is also associated with cataract formation [1, 33], which is easy to observe in mice [34], but was not seen in our mice. XFM may accumulate on the cornea [1, 35], which is also readily imaged in mice [34, 36, 37], but was not seen in our mice. In our experience, foreign substances in the anterior chamber of mice typically cause a mobilization of macrophages, which we are experienced at observing [38, 39], but did see in any of our mice. Likewise, in our experience, glaucomatous elevations in IOP are sometimes accompanied by buphthalmia [34, 40], however, enlarged eyes were not observed in our cohorts of mice. In sum, there are additional assays and observation techniques that might have added to this analysis, but the overall results of this primary screen are that the anterior segments in our new mutant mouse lines are normal in appearance and lacking overt primary or secondary indices of XFS.

Though not overtly resembling human eyes with XFS, *Loxl1* mutant strains did exhibit several abnormal ocular and non-ocular phenotypes. LOXL1 functions in the biosynthesis and maturation of connective tissue [41]. Aside from pelvic organ prolapse, which is an overt disease of connective tissue, some of the observed ocular phenotypes were also suggestive of connective tissue disease. Amongst these, the unusual phenotype observed in the temporal conjunctiva is perhaps the one with greatest possible link to XFS. The conjunctiva is rich in elastin [42–44], and though less dramatic in presentation, human XFS also includes conjunctival abnormalities [1, 45]. The phenotype observed in the mice involved an apparent laxity in the temporal bulbar conjunctiva. *Loxl1* mutant homozygotes also exhibited a previously noted decrease in CCT [20]. XFS has been associated with decreased CCT in some [46–48], but not all human studies [49, 50]. Decreased CCT is a feature of other disorders of connective tissue, such as Marfan and Loeys-Dietz syndromes [51, 52]. Mutant homozygotes also had decreased GCC thickness, which can also occur in Marfan syndrome [53], and increased ACD.

Aside from the phenotypes of *Loxl1* mutants, mice from the other strains exhibited few abnormal phenotypes, which were of modest or no known relevance to XFS. *Sema6a* mutation led to increases in both GCC thickness and RT, of nearly the same degree, suggesting that the difference in RT was due to the difference in GCC thickness. This thickening, as well as the coarse appearance of the GCC in SD-OCT imaging, likely correlates to a previously identified defect in dendritic stratification of starburst amacrine cells [54]. Of note, one of the three *Sema6a* mutant strains (*Sema6a^em3Andm^*, an in-frame deletion of 4 amino acids) did not exhibit an increase in RT or GCC, indicating that this variant was a hypomorph in comparison to the others. As predicted from other studies, *Agpat1* and *Cacna1a* mutation both led to early lethality of homozygotes, with several defects noted in the few *Cacna1a* homozygotes examined, but no ocular phenotypes were detected among heterozygotes for either gene. A difference in RT was detected among the *Rbms3* cohorts, but only in a specific time and cohort comparison (mutant to heterozygote).

To date, large genetic association studies in humans have identified seven genome-wide significant XFS susceptibility loci (*P* < 5 × 10^−8^) associated with common SNPs (6p21, 19p13.13, 15q24.1, 13q12, 3p24, 5q23, and 11q23.3) [13–15] and one locus associated with rare variants [16]. Smaller studies have suggested additional loci with possible significance in at least some populations [55, 56]. The specific functional mutations that are the source of XFS risk in each of these loci have not been established. However, a combination of prioritization rationales based on SNP location, gene expression, and pathway analysis have pointed to *AGPAT1*, *CACNA1A*, *LOXL1*, *POMP*, *RBMS3*, *SEMA6A*, and *TLCD5* (also referred to as *TMEM136*) as the most likely candidates for the common signals. In some of the loci, there is evidence of both damaging and protective alleles [13]; and for all of them, most of the associated SNPs are non-coding and presumed to act via transcriptional regulation of a nearby gene [13].

The challenge of defining effectors from GWAS data is widespread. Among attempts to study GWAS-identified loci from human populations in mice, there are examples of successes using mice in which genome manipulations have confirmed the identity of a candidate gene by recapitulating the human phenotype. These manipulations have included knock-in of coding SNPs [57], deletion of conserved sequences flanking SNPs in regulatory regions [58], knockouts of protein coding genes [59], transgenic overexpression of candidates [60], and deletions of lncRNAs [61]. However, there are also examples of experiments with mice that failed to confirm an expected candidate [62] or in which more than one gene in a GWAS-identified locus was found to influence the relevant phenotype [63]. One of the main challenges is that both intergenic and intragenic SNPs can influence the transcript levels of genes other than the nearest one—sometimes at Mb distances [64–69]. To deal with this complexity, some recent approaches have taken a multi-step approach in which high-throughput CRISPR screens are first utilized to define *cis*-regulatory elements overlapping GWAS variants regulating gene expression [70, 71], and these results are secondarily integrated with in vivo models such as mice [71]. In this approach, a plausible mechanism for many GWAS loci can be refined, but some studies have found that > 50% of GWAS loci do not have an apparent target gene in the immediate proximity (within 500 kb) [70]. In considering GWAS loci for XFS/XFG in the context of our current findings, we suggest that the logical strategies would be to better define *cis*-regulatory regions with target genes in each locus (in case the effectors are not the nearest gene) and to also consider over-expression manipulations.

There have been previous reports of mouse models with partial similarities to human XFS [8]. Two examples include *Lyst^bg-J^* mice [31] and mice injected with an adenovirus transiently expressing *Wnt5a* [72]. Both models include variations of XFS-like iris defects and microscopic deposits of XFM-like material. However, neither *Lyst* nor *Wnt5a* are genes with human genetic associations to XFS, neither model has been shown to develop glaucoma, and the accumulation of XFM-like deposits in both models is modest. Another model of relevance are transgenic mice overexpressing *Loxl1* from the βB1-crystallin promoter element [73]. These mice exhibit an aberrant aggregation of insoluble LOXL1 in lens fibers and on the extracellular lens capsule. These findings suggest that an intracellular aggregation of LOXL1, followed by its secretion, may have a role in early XFS pathology. However, this model does not show accumulation of XFS-like material and the eyes maintain an overtly healthy appearance. There have been multiple studies of *Loxl1* knockout mice, which have variably observed abnormalities in the blood:aqueous barrier, lens abnormalities, altered conventional outflow physiology, and altered biomechanical properties of the peripapillary sclera—but not XFM accumulation [20, 74, 75]. Thus, each of the existing models has allowed some progress, but none have the most clinically recognized features of XFS—robust accumulations of XFM visible by slit-lamp that led to glaucoma.

The large number of mice involved in the screen and concurrence of these experiments with the COVID-19 pandemic resulted in some caveats to this study that merit mention. First, the screen was limited by necessity to a single genetic background (C57BL/6J) and did not include extreme aging. A model on this widely used background with readily identifiable XFM at age under 1 year of age would have been the ideal model needed by the field. However, genetic background and age can influence every phenotype and it is possible that other combinations of these factors could have yielded different results. Of note, the relevant XFS-like iris defects of *Lyst* mutant mice can be observed, to various degrees, on both the C57BL/6J and DBA/2J genetic backgrounds [30, 31]—supporting that the C57BL/6J background is at least partially permissive to XFS phenotypes. Second, the screening primarily relied on slit-lamp and SD-OCT exams. Although we designed our screen to identify a mouse model with overt XFM visible in the anterior segment that would facilitate future studies oSf XFS, it is possible that some strains contained microscopic traces of XFM in the eye or that there were systemic phenotypes that were unnoticed. Third, we prioritized variants predicted to be deleterious, mostly deletions and premature stop codons, but we did not conduct molecular assays to study the variants in depth. Given that non-ocular phenotypes of the current strains matched those of knockouts (for the strains in which knockouts have been published; pelvic organ prolapse of *Loxl1* mutants [18–20], wobbly gait and early lethality of *Cacna1a* [22, 23], and small body size and early lethality of *Agpat1* [24]) and that we studied multiple alleles for some of the strains, it is reasonable to assume that most were uncomplicated loss-of-function mutations, but this uncertainty is nonetheless a caveat.

In sum, this study has conducted a screen in mice for a model of XFS based on manipulation of genes from GWAS loci identified in human studies. Despite some caveats, the combined results indicate that monogenic loss-of-function mutations in these genes will not yield a robust mouse model of XFS on the C57BL/6J background. It is unclear if these results reflect a complexity of modeling human disease with mice, complexity of the GWAS loci, or multi-factorial genetic and environmental complexity of the disease. At a minimum, it can be concluded that future attempts to create an animal model of XFS based on manipulation of genes from these GWAS loci will need to attempt different strategies.

## Materials and methods

### Genome manipulations in mice

All animals were treated in accordance with the ARVO Statement for the Use of Animals in Ophthalmic and Vision Research. All experimental protocols were approved by the Animal Care and Use Committee of the University of Iowa. Targeting to produce variants in the *Agpat1*, *Cacna1a*, *Loxl1*, *Pomp*, *Rbms3*, *Sema6a,* and *Tlcd5* genes was conducted by the University of Iowa Genome Editing Facility. Using C57BL/6J mice, individual variants were generated using CRISPR/Cas9 nuclease in combination with target-specific gRNAs (Supplemental File 8). Following injection, targeting events were screened by PCR followed by Sanger sequencing of genomic DNA from founders (Supplemental File 9). CRISPR/Cas9-mediated genetic variants predicted to cause frameshifts, altered splicing, or potentially significant deletions were selectively maintained. Founder mice were bred to C57BL/6J mice to confirm germline transmission and the targeted allele was confirmed by Sanger sequencing. Each colony was expanded to establish experimental cohorts consisting of N_2-3_F_2–4_ mice. Ear punches from all mice used in the study were sent to Transnetyx (Cordova, TN) for genotyping using real-time PCR.

### Experimental cohorts

From the combined strains, mice were assigned into one of two experimental cohorts (Supplemental File 10). Cohort 1 was generated for successive imaging at “Early” (~1–2 month) and “Mid” (~4–5 month) time points. The experimental design for this cohort was to systematically screen both eyes of multiple males and females of homozygous mutant, heterozygote, and homozygous wild-type mice for indices of exfoliation or other ocular phenotypes. Cohort 1 ultimately included 224 mice from 12 strains and was studied by successive slit-lamp exams at Early (average actual age 36 days) and Mid (average actual age 122 days) time points. Eyes were also screened systematically by successive SD-OCT imaging at Early (average actual age 41 days) and Mid (average actual age 131 days) time points, and some mice of all strains screened by single fundus exams prior to euthanasia (average actual age 149 days). Most individual mice were examined at both time points, but there were exceptions, and the study design was to emphasize imaging of as many eyes as possible with no data exclusion criteria. Thus, the number of exams per age and genotype are not equal across groups. A second, independent, group (“Cohort 2”) was generated for sampling the effects of extensive aging at an “Old” (~12 month, average actual age 379 days) time point. The experimental design for this cohort was to screen both eyes of females, which were predominantly heterozygotes, co-housed in 1–2 pens per strain. A small number of homozygotes were also included in this cohort, including 5 *Tlcd5* mutants (all female) and 4 *Sema6a* mutants (all female). Due to a highly penetrant phenotype of pelvic organ prolapse amongst *Loxl1*-mutant homozygotes, it was realized during the study that it would be impractical to age homozygotes extensively. To compensate, we revised the plan for this genotype to attempt aging mice intended for both cohorts to the oldest age feasible, which came to involve two mice studied at 181 and 190 days of age. Cohort 2 ultimately included 53 total mice from 10 strains and was studied by single time point SD-OCT, slit-lamp, and fundus exams.

### Slit-lamp examination

Anterior chamber phenotypes were assessed in conscious mice, using a slit-lamp at 25X (SL-D7; Topcon, Tokyo, Japan), and photodocumented using a digital camera (D800; Nikon, Tokyo, Japan). Unless specifically noted, all photographs were taken with identical slit-lamp settings and documented using identical camera settings and image processing (the only exception being panel E of Supplemental File 7, which used higher magnification and post-processing adjustments to brightness to accentuate a very small feature). For every mouse, both eyes were examined by broad beam and transilluminating illumination, and visually inspected. Broad beam photographs were collected for every eye and transillumination images were collected only for the left eye. Because the conjunctival phenotype of *Loxl1* mutants was not recognized until the overall study was midway to completion, photographs of the medial and lateral canthus were only collected after its relevance became recognized (458 of the 902 slit-lamp exams, including 112 exams of mice homozygous for *Loxl1* mutation).

### SD-OCT imaging

Animals were injected with a standard mixture of ketamine/xylazine (intraperitoneal injection of 100 mg ketamine +10 mg xylazine/kg body weight; Ketaset®, Fort Dodge Animal Health, Fort Dodge, IA; AnaSed®, Lloyd Laboratories, Shenandoah, IA). During induction of anesthesia, the mice were provided supplemental indirect warmth by a heating pad. Immediately upon anesthesia, eyes were hydrated with balanced salt solution (BSS; Alcon Laboratories, Fort Worth, TX) and SD-OCT images obtained (Bioptigen, Inc., USA). For anterior segment imaging, a 12-mm telecentric bore was positioned such that the pupil of the eye was centered in the volume intensity projection. Scan parameters were as follows: radial volume scans 2.0 mm in diameter, 1000 A-scans/B-scan, 100 B-scans/volume, 1 frame/B-scan, and 1 volume. For retinal imaging, the mouse retina bore was positioned such that the optic nerve of the eye was centered in the volume intensity projection. Scan parameters were as follows: rectangular volume scans 1.4 mm × 1.4 mm in size, 1000 A-scans/B-scan, 100 B-scans/volume, 1 frame/B-scan, and 1 volume. Following imaging, eyes were hydrated with artificial tears and mice were provided supplemental indirect warmth for anesthesia recovery. Using the Bioptigen InVivoVue computer software, central corneal thickness (CCT) was measured for each eye with vertical angle-locked B-scan calipers. Mice were included in the analysis if the standard deviation (SD) between the right and left corneas was less than 5 μm, and if both eyes were free from opacity. Anterior chamber depth (ACD) was measured for each eye with vertical angle-locked B-scan calipers from the posterior central cornea to the anterior of the lens. Mice were included in the analysis if the SD between the right and left ACD was les that 25 μm, and if both eyes were free from opacity. Retinal thickness (RT) and retinal ganglion cell complex (GCC) thickness were also measured for each eye with vertical angle-locked B-scan calipers. Mice were included if both eyes were free from opacity. All images were processed identically.

### Fundus imaging

The pupils of mice were dilated using a combination of 2% cyclopentolate hydrochloride ophthalmic solution (Cyclogyl®, Alcon Laboratories, Fort Worth, TX) and 2.5% phenylephrine hydrochloride ophthalmic solution (Paragon BioTeck, Inc., Portland, OR). When pupils were fully dilated, mice were injected with a standard mixture of ketamine/xylazine (intraperitoneal injection of (100 mg ketamine +10 mg xylazine)/kg body weight (Ketaset®, Fort Dodge Animal Health, Fort Dodge, IA; AnaSed®, Lloyd Laboratories, Shenandoah, IA). Mice were provided supplemental indirect warmth by a heating pad during anesthesia. Immediately upon anesthesia, hypromellose 2.5% ophthalmic demulcent solution (Goniovisc®, HUB Pharmaceuticals, LLC, Rancho Cucamnoga, CA) was applied to each eye. Eyes were imaged with a Micron III retinal imaging microscope (Phoenix Research Labs, Pleasanton, CA). Following imaging, eyes were hydrated with artificial tears and mice were provided supplemental indirect warmth for anesthesia recovery. All images were processed identically.

### Statistical analysis

Three approaches were used. 1) Pairwise comparison. For all strains, quantitative ocular differences were evaluated using Student two-tailed *t*-test in a pairwise fashion between independent genotypes within each individual strain. 2) ANOVA comparison. For strains with three genotypes available for comparison, differences were evaluated using one-way ANOVA with Tukey post-test. 3) Binned comparison. For contexts in which multiple different alleles were available, data from multiple strains were fused into 3 bins (all homozygous wild-type, all heterozygous, all homozygous mutant) prior to analysis with one-way ANOVA with Tukey post-test. For the *Loxl1* strains, this included mice from all 5 strains; for the *Sema6a* strains this included mice from the two strains that had a similar distinctive retinal appearance in SD-OCT imaging. In all approaches, sample groups had n ≥ 4 animals and there were no corrections for multiple comparisons.

### Data sharing

The full dataset of slit-lamp images (.jpg) contributing to this study are available through Iowa Research Online at the University of Iowa (doi: 10.25820/data.006799) [17]. The data are organized into compressed sub-folders based on candidate gene, which can be downloaded and decompressed to view additional levels of folder organization based on allele-genotype-age-mouse ID. Data tables (.csv) and explanations of file organization (.txt) are also available. These freely available data will be maintained in this format for at least 10 years from the date of publication.

## Supplementary Material

Supplemental_File_1_Slit-lamp_data_ddae088

Supplemental_File_2_ddae088

Supplemental_File_3_Individual_SD-OCT_data_ddae088

Supplemental_File_4_Group_SD-OCT_data_ddae088

Supplemental_File_5_ddae088

Supplemental_File_6_ddae088

Supplemental_File_7_ddae088

Supplemental_File_8_Targeting_Strategies_ddae088

Supplemental_File_9_Mutations_ddae088

Supplemental_File_10_Cohorts_ddae088

Supplemental_Figure_Legends_ddae088
