# Supplementary figures and images for: Lack of evidence for GWAS signals of exfoliation glaucoma working via monogenic loss-of-function mutation in the nearest gene
Source: Hum Mol Genet. 2024 May 20;33(17):1481–94. doi: 10.1093/hmg/ddae088 (PMC13142156; doi:10.1093/hmg/ddae088)

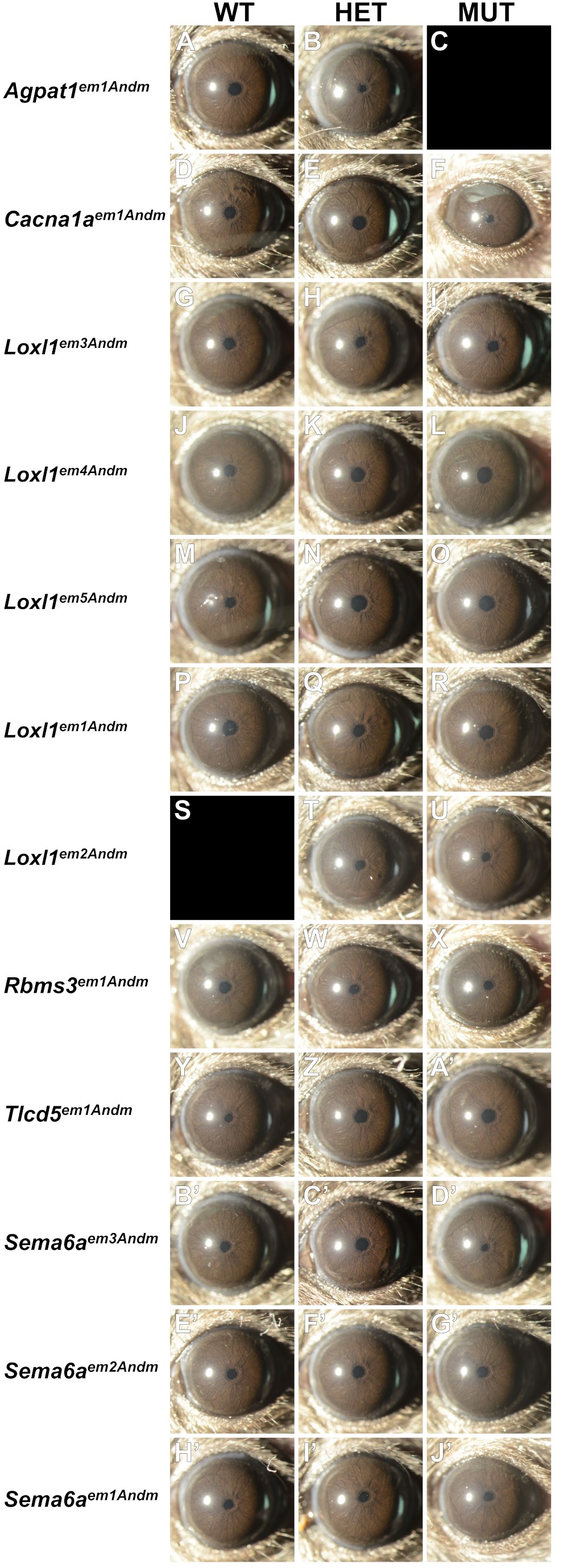

Supplement: Supplemental_File_2_ddae088 [file supplemental_file_2_ddae088.jpeg]

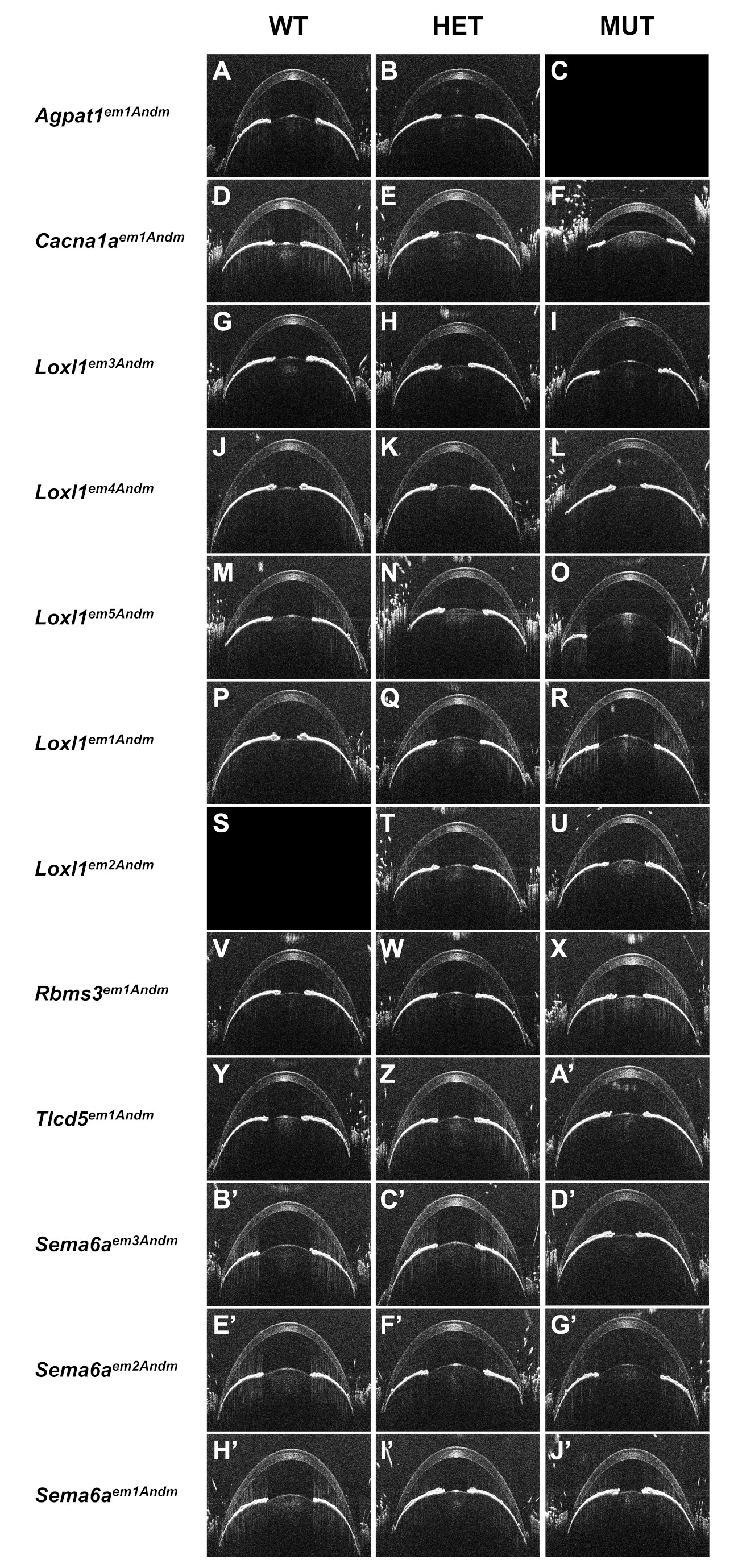

Supplement: Supplemental_File_5_ddae088 [file supplemental_file_5_ddae088.jpeg]

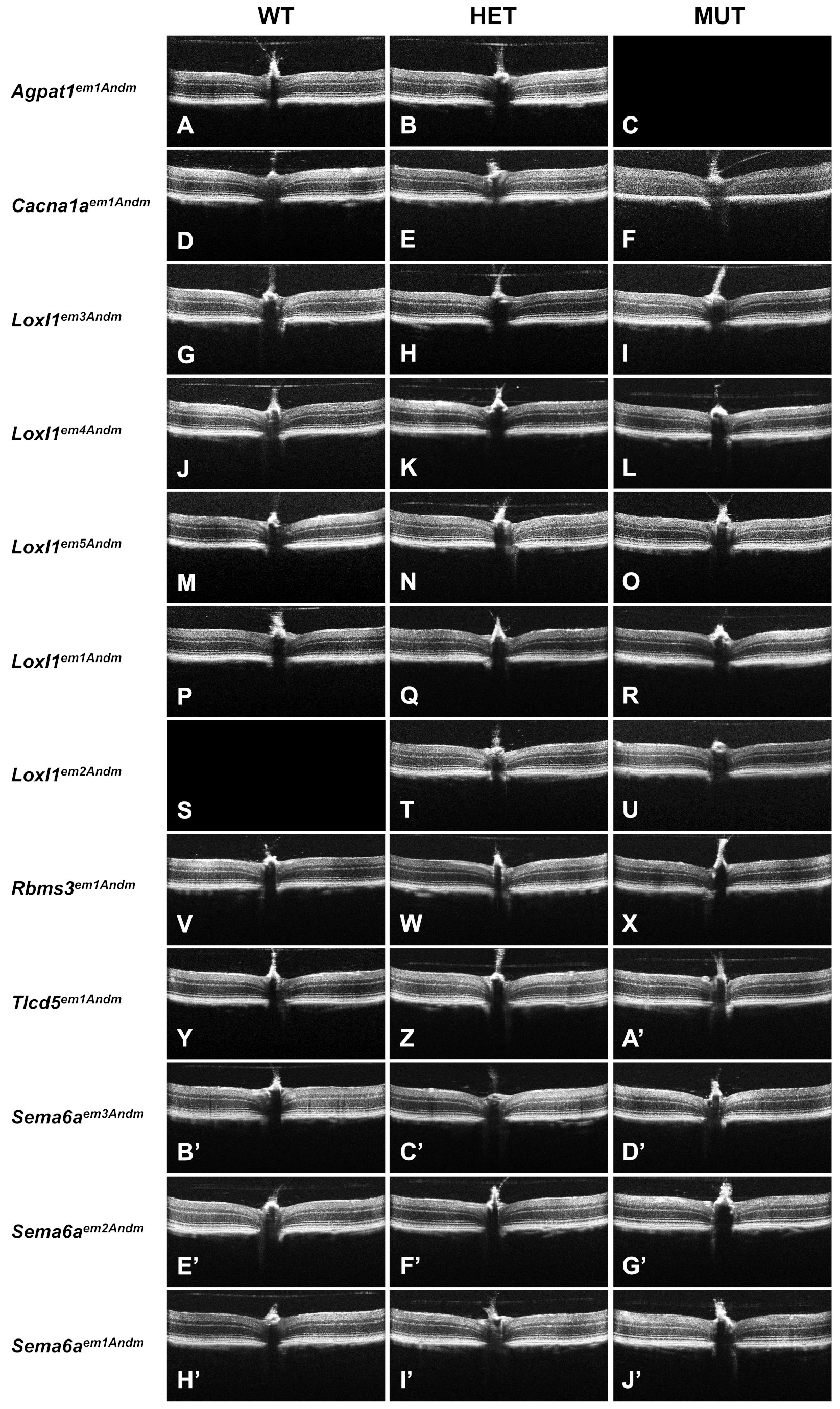

Supplement: Supplemental_File_6_ddae088 [file supplemental_file_6_ddae088.jpeg]

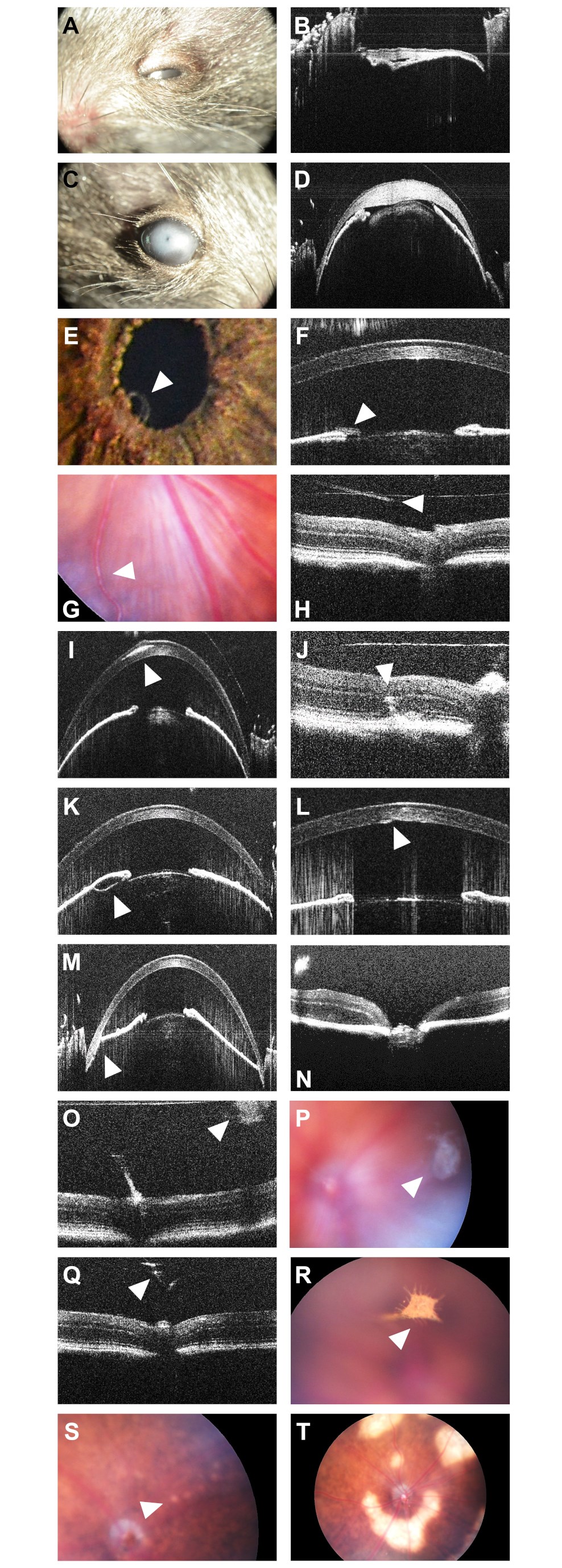

Supplement: Supplemental_File_7_ddae088 [file supplemental_file_7_ddae088.jpeg]
