# Supplementary material for: Lack of evidence for GWAS signals of exfoliation glaucoma working via monogenic loss-of-function mutation in the nearest gene
Source: Hum Mol Genet. 2024 May 20;33(17):1481–94. doi: 10.1093/hmg/ddae088 (PMC13142156; doi:10.1093/hmg/ddae088)
Supplement: Supplemental_File_8_Targeting_Strategies_ddae088 [file supplemental_file_8_targeting_strategies_ddae088.pptx]

## Slide 1
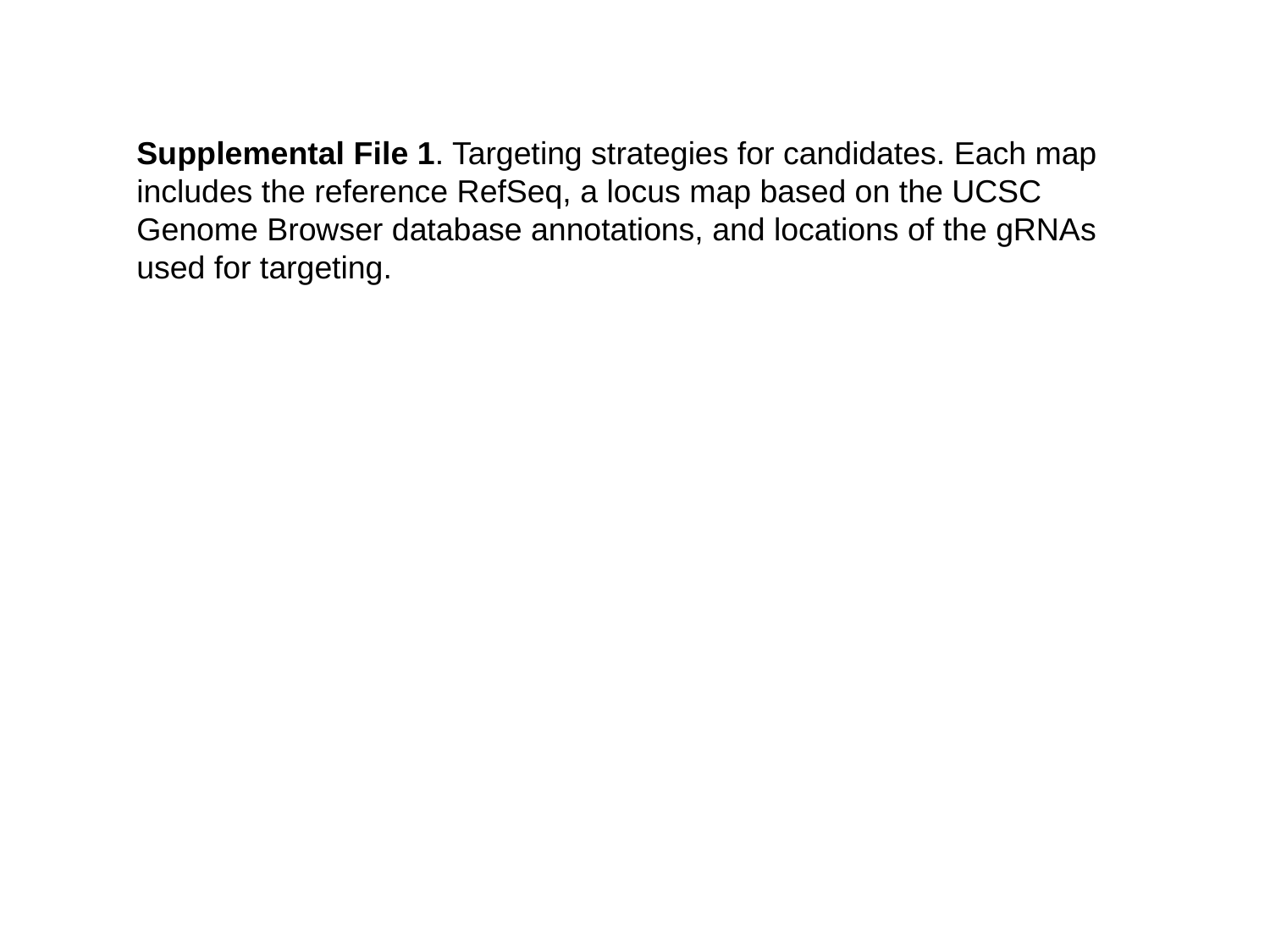

Supplemental File 1. Targeting strategies for candidates. Each map includes the reference RefSeq, a locus map based on the UCSC Genome Browser database annotations, and locations of the gRNAs used for targeting.

## Slide 2
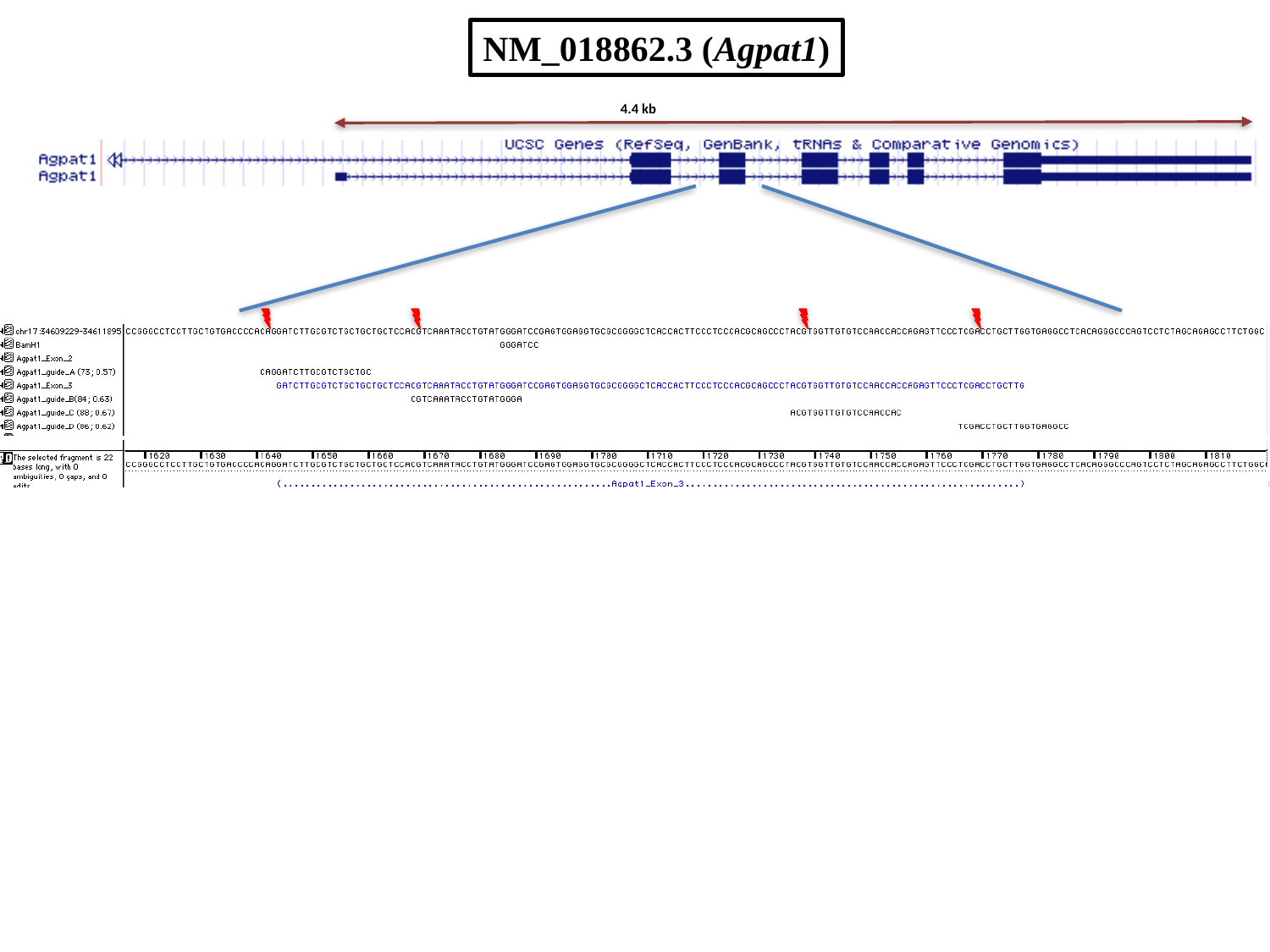

NM_018862.3 (Agpat1)
4.4 kb

## Slide 3
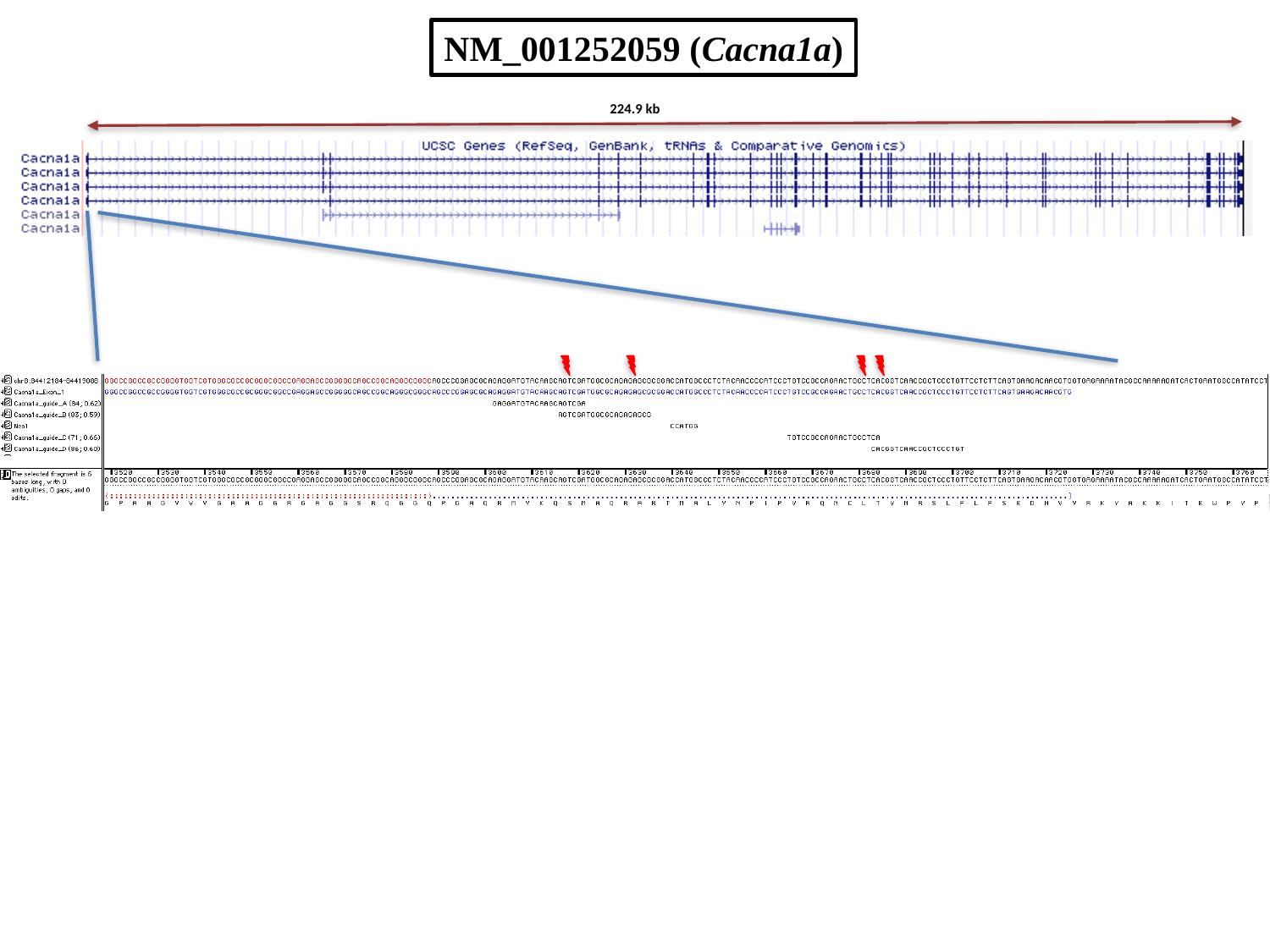

NM_001252059 (Cacna1a)
224.9 kb

## Slide 4
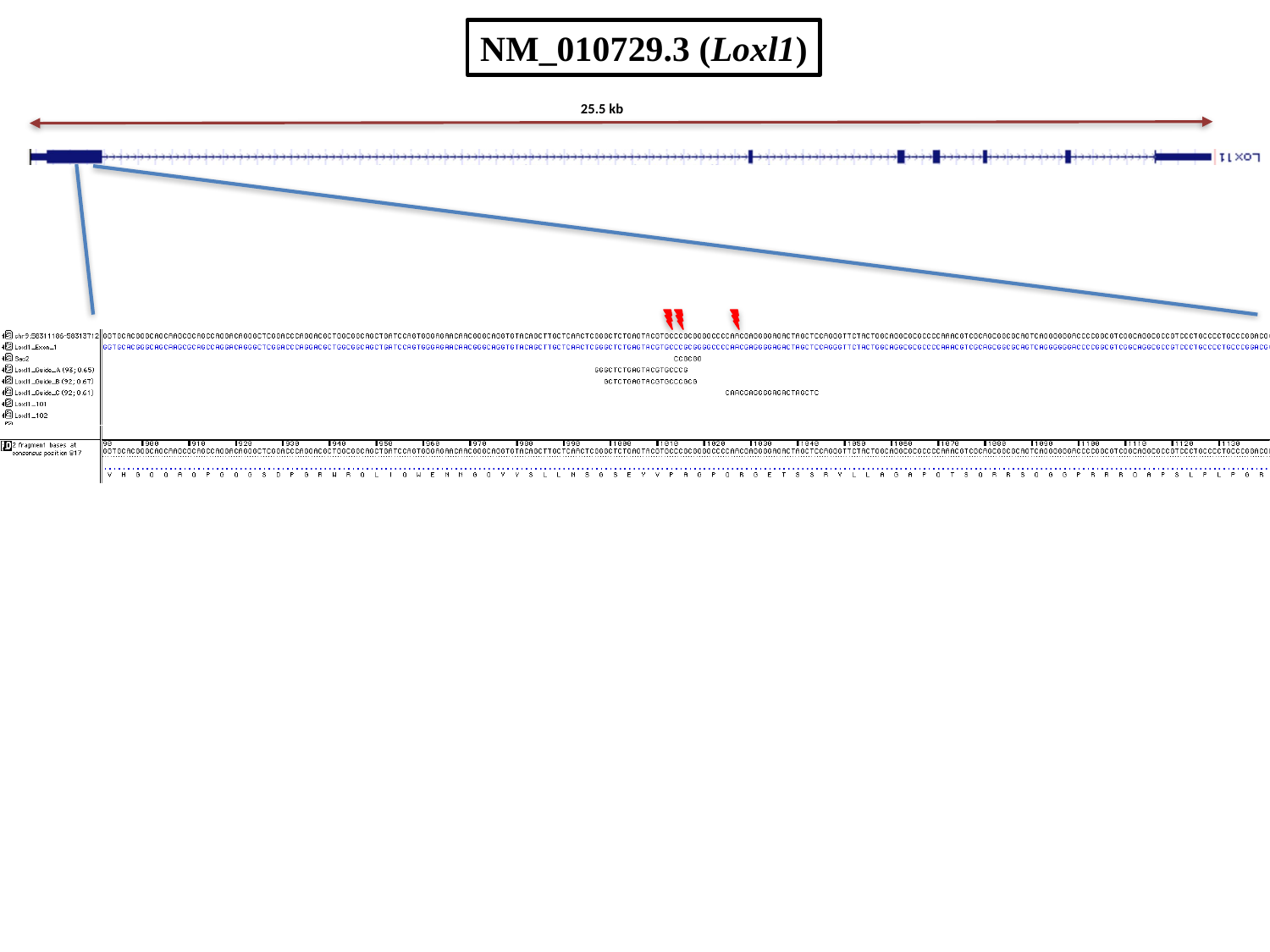

NM_010729.3 (Loxl1)
25.5 kb

## Slide 5
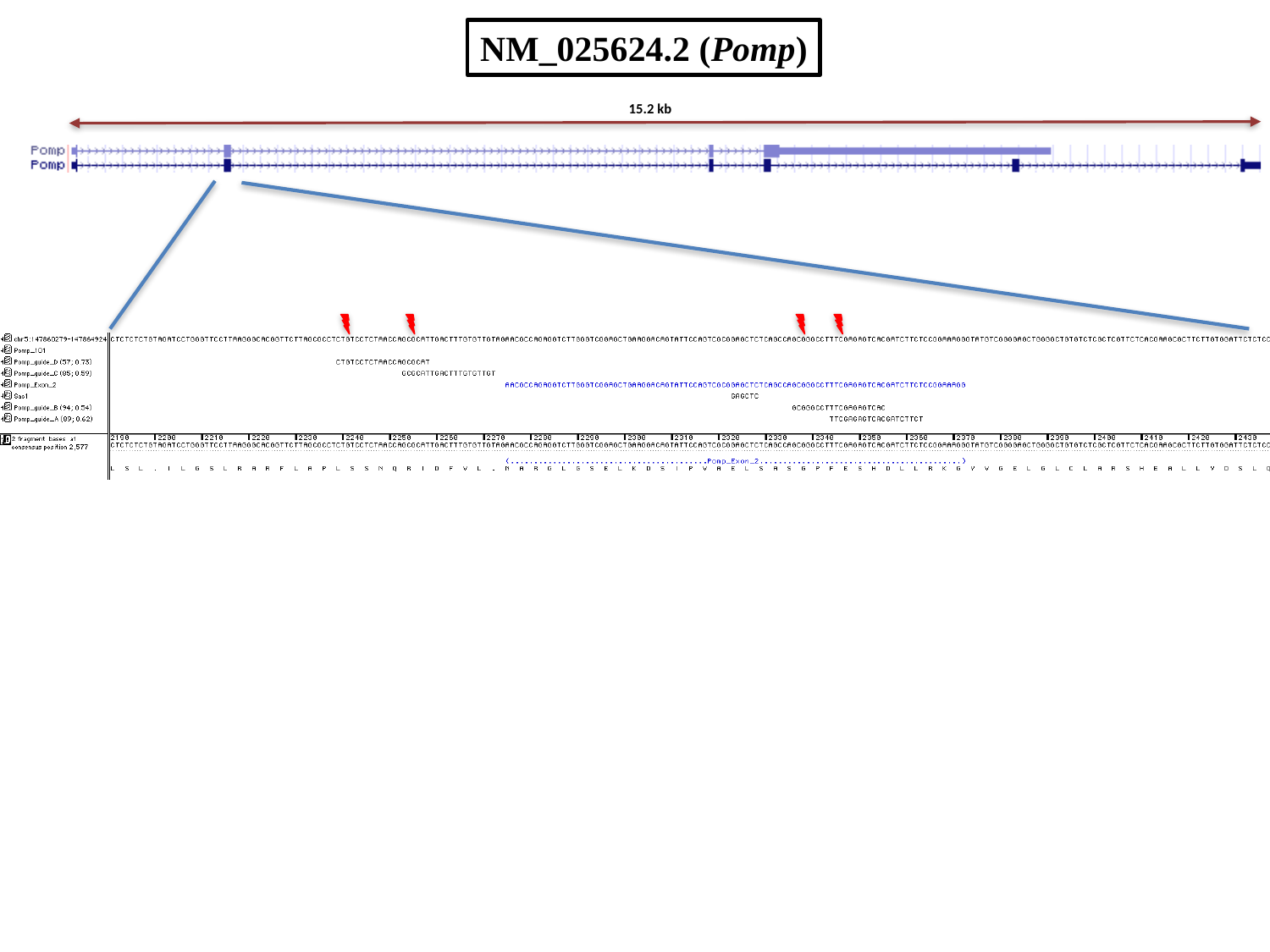

NM_025624.2 (Pomp)
15.2 kb

## Slide 6
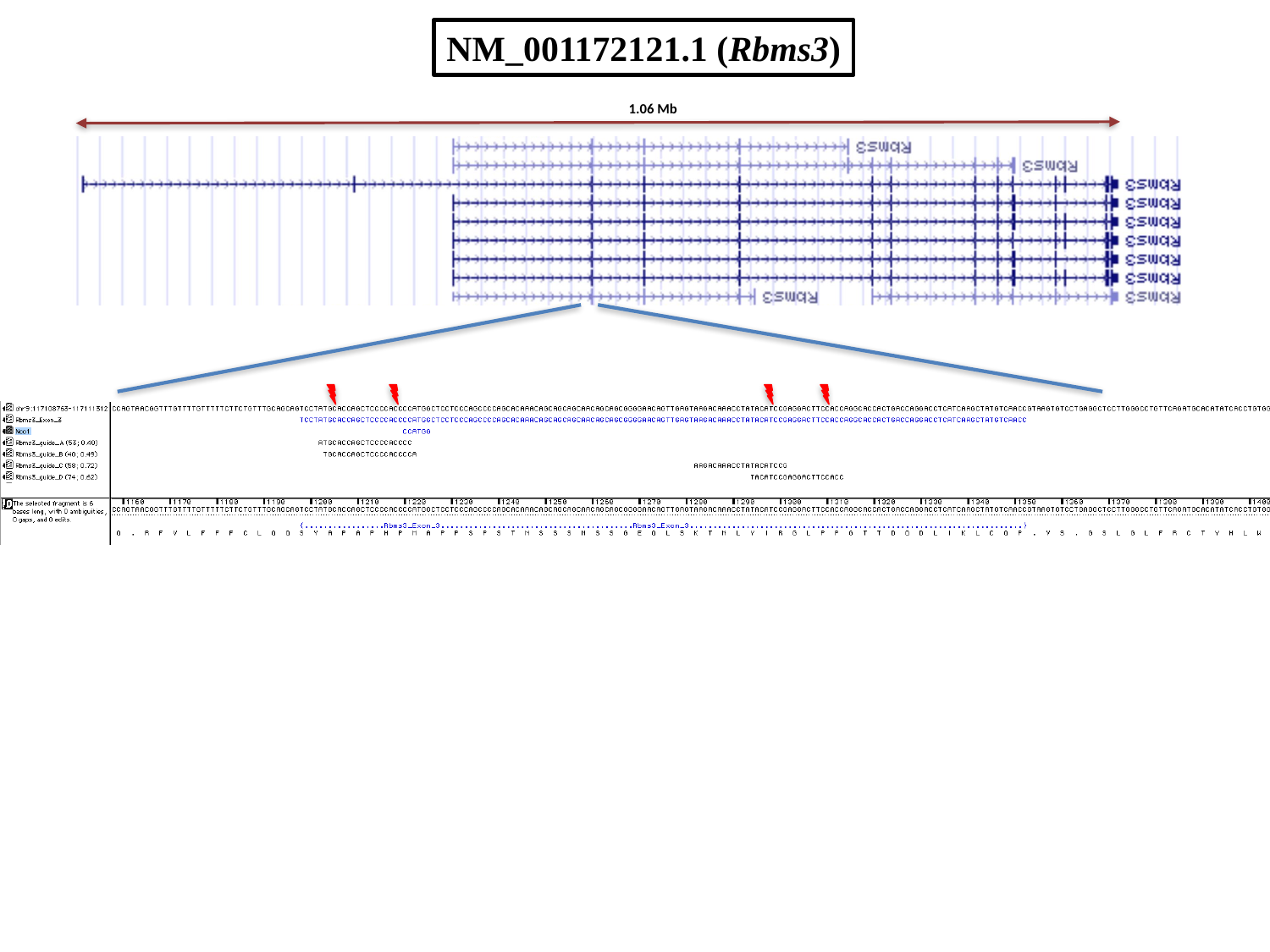

NM_001172121.1 (Rbms3)
1.06 Mb

## Slide 7
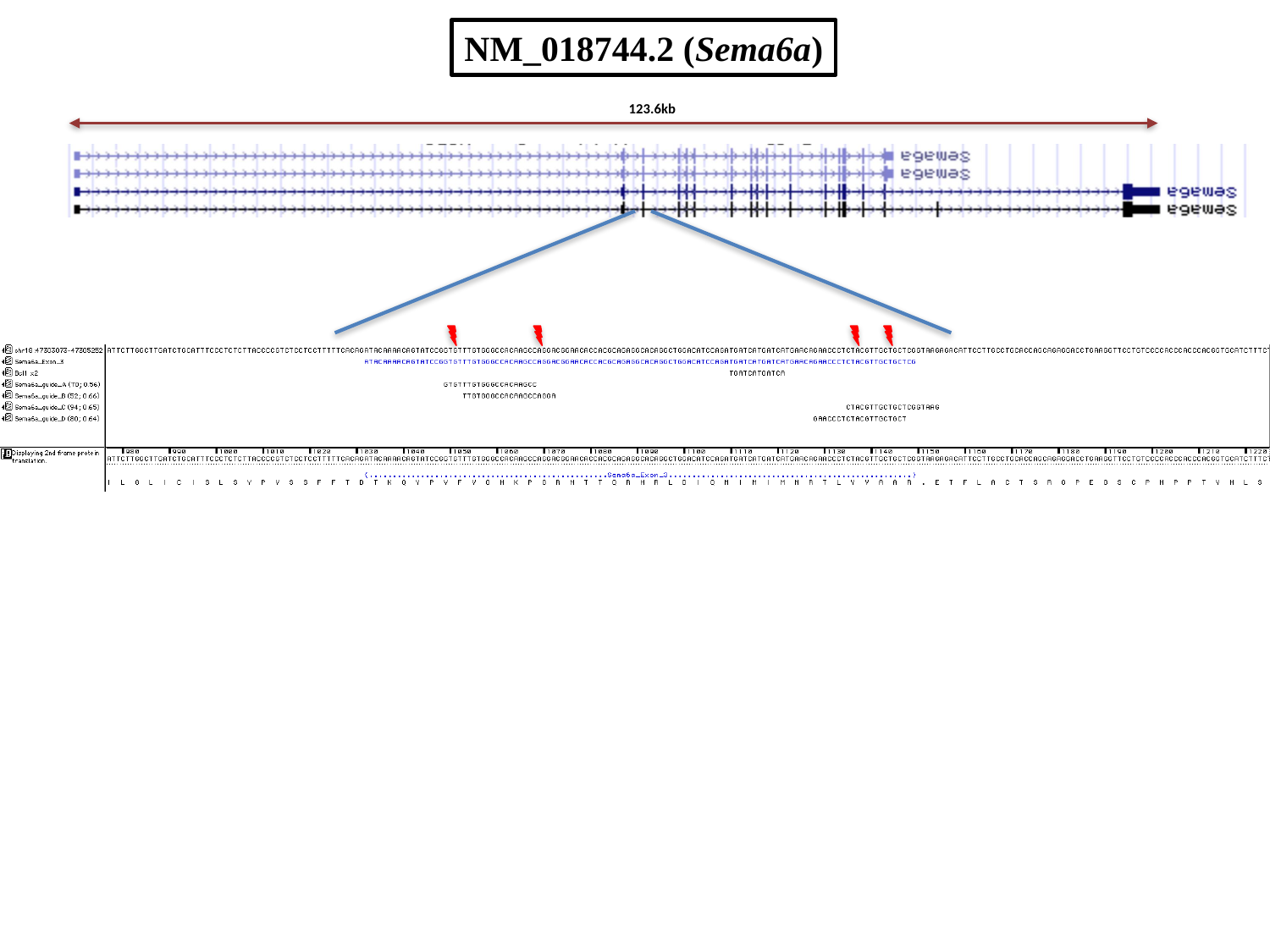

NM_018744.2 (Sema6a)
123.6kb

## Slide 8
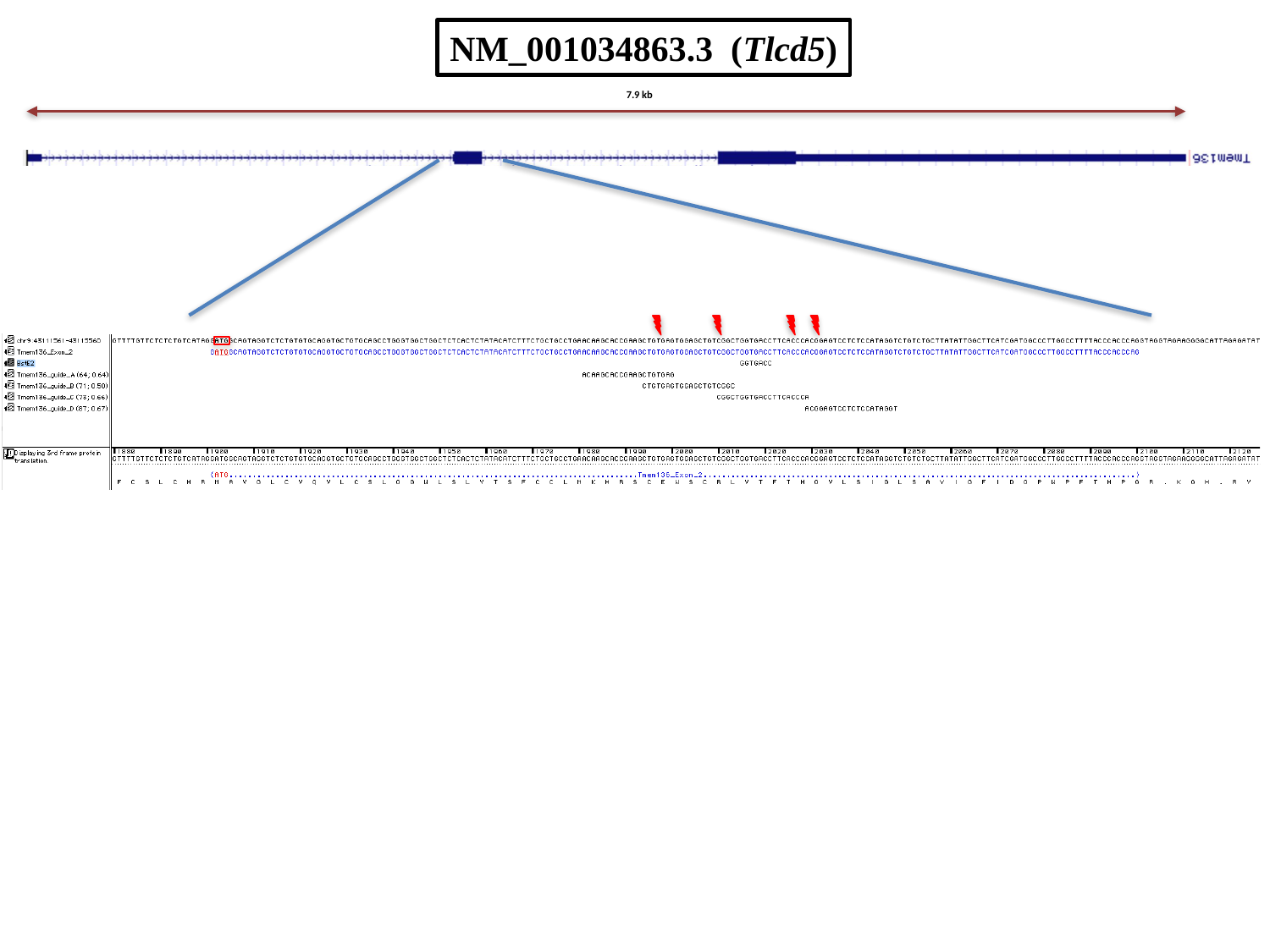

NM_001034863.3 (Tlcd5)
7.9 kb
