# Supplementary material for: Lack of evidence for GWAS signals of exfoliation glaucoma working via monogenic loss-of-function mutation in the nearest gene
Source: Hum Mol Genet. 2024 May 20;33(17):1481–94. doi: 10.1093/hmg/ddae088 (PMC13142156; doi:10.1093/hmg/ddae088)
Supplement: Supplemental_File_9_Mutations_ddae088 [file supplemental_file_9_mutations_ddae088.docx]

**Supplemental File 2.** Detailed descriptions of mutations. Sequences shown from UCSC Genome Browser (GRCm39/mm39). Based on UCSC Annotation of RefSeq. Showing either the affected exon and 100 bp of flanking intronic sequences.

***Agpat1* (NM_018862.3)**

Showing exon 3 and 100 bp of flanking introns

Yellow highlight = deleted

Capital case = exon

***Agpat1^em1Andm^* (175-bp del):** 175-bp deletion encompassing 116 bp of coding sequence, splice donor of the targeted exon, and 59 bp of intron. Predicted to disrupt splicing.

>mm39_refGene_NM_018862_4 range=chr17:34829746-34830079 5'pad=100 3'pad=100 strand=+ repeatMasking=none

atacagaggcggccagaatgcaaggaataggtagcgggtatggaggttgg

catgtggacgccagagaatcccaccgggcctccttgctgtgaccccacag

GATCTTGCGTCTGCTGCTGCTCCACGTCAAATACCTGTATGGGATCCGAG

TGGAGGTGCGCGGGGCTCACCACTTCCCTCCCACGCAGCCCTACGTGGTT

GTGTCCAACCACCAGAGTTCCCTCGACCTGCTTGgtgaggcctcacaggg

cccagtcctctagcagagccttctggcccctcccatacctgcttttccct

cctcttggatgtcatctgtcctcaggcctccttc

***Cacna1a* (NM_001252059)**

Showing exon 1 and 100 bp of flanking introns

Red text = inserted

Capital case = exon

***Cacna1a^em1Andm^* (1-bp ins):** 1-bp coding sequence insertion in the targeted exon. Predicted to cause frameshift and premature STOP in the targeted exon.

>mm39_refGene_NM_001252059_0 range=chr8:85141893-85142637 5'pad=100 3'pad=100 strand=+ repeatMasking=none

ggtggccggggatcgcctggatcgcgtccccggttatttttgccgcgccc

ctcccccgcgccccgcccccgcgccctcccccgcacccctcctcccgccc

GGGGTCACCGCCTCTGCCGGCATGTCCCGAGCCGCGCTCCCCGGCTCGGC

CGGGCCGTCCCGCTCGTGAGCCCCCCGAGCTGAGGCCGGGCGCCGCCGCC

CGATGGGCTGGGCCGTGGAGCGTCTCCGCAGTCGCAGGTGCAGCCGCCGT

CCCCACAGCCCGGCCAGCCTGAGCAGCGGCGGCGGTGGTGGTGGTGGTGG

TGGCGGTGGCCGTGGCTTCCGCAGCGCTCGCCGCAGCAACAGCAGCCGCC

GCGTAACCCGAGGCCCTTTGCTCTTTGCAGAATGGCCCGCTTTGGAGACG

AGATGCCGGGCCGCTACGGCGCAGGCGGAGGAGGCTCAGGGCCGGCCGCC

GGGGTGGTCGTGGGCGCCGCGGGCGGCCGAGGAGCCGGGGGCAGCCGGCA

GGGCGGGCAGCCCGGAGCGCAGAGGATGTACAAGCAGTCGATGGCGCAGA

GA **C** GCGCGGACCATGGCCCTCTACAACCCCATCCCTGTCCGCCAGAACTGC

CTCACGGTCAACCGCTCCCTGTTCCTCTTCAGTGAAGACAACGTGgtgag

aaaatacgccaaaaagatcactgaatggccatatccttttgccccagccc

gagcggctgcgcctcccctcctcctcttcttcctcctcttcttct

***Loxl1* (NM_010729.3)**

Showing exon 1 and 100 bp of flanking introns

Red text = inserted

Yellow highlight = deleted

Capital case = exon

***Loxl1^em1Andm^* (41-bp del):** 41-bp coding sequence deletion in the targeted exon. Predicted to cause frameshift and premature STOP in the targeted exon.

>mm39_ncbiRefSeqCurated_NM_010729.3_0 range=chr9:58218869-58220595 5'pad=100 3'pad=100 strand=- repeatMasking=none

ggacaggccacatagctaaaacccggcgggccgtagggccccgcggagga

ggccccagcaggcggaccaggtttctgaaacctccagacactttcagcct

GTTGCTTATTCATTCAGAGTGGGAAAGCGCCAGCCTAGCGAGCAGCCAGC

CAGTGTGGGGCTGGCCATGTAAGGGCCCCAGGAGGTCCTGCCCGTCCCGT

GCCCTACGGAAAGCCTCGTGCAGCCCTGGGCACCGCCCCTGCCCTGCCCT

GACCCCTTGGCCTCGAAATGCTGTCATCGGAGGAGCCGTCCCGCTCGGGA

CAAGGCCAGGATGGACAAAGCTAGAGCTTGGGCAGGCGAAGAGCCATCCT

GTCCTCGAGGCCGTGGGAAGAGAAGCACGCACCGAGGCCCACTCTCTGCC

CACCCGGCCTCTGCAGAAGGGCCACTATGGCTCTGGCCGGAGCCGGCAGC

CAGCTGAGGACCCTGGTGTGGAGTGCCTGCCTGTGCGTCCTGGTGCACGG

GCAGCAAGCGCAGCCAGGACAGGGCTCGGACCCAGGACGCTGGCGGCAGC

TGATCCAGTGGGAGAACAACGGGCAGGTGTACAGCTTGCTCAACTCGGGC

TCTGAGTACGTGCCCGCGGGGCCCCAACGAGGGGAGACTAGCTCCAGGGT

TCTACTGGCAGGCGCGCCCCAAACGTCGCAGCGGCGCAGTCAGGGGGGAC

CCCGGCGTCGGCAGGCGCCGTCCCTGCCCCTGCCCGGACGCGTGGGTTCG

GACACTGTGCGCGGCCAAACGCGGCACCCGTTCGGCTTCGGCCAGGTGCC

CGACAACTGGAGAGAGGTGGCCGTCGGGGACAGTACCGGCATGGCTCGGG

CCCGCACCTCCGTCTCCCAGCAACGGCACGGGGGCTCCGCCTCCTCCTCG

GTTTCGGCCTCGGCCTTCGCCACCACCTATCGCCAGCCATCCTCCTACCC

GCAGCAGTTCCCCTATCCGCAGGCGCCCTTCGTAAACCAGTATGAGAACT

ACGATCCCGCGTCACGGACCTACGAACAGGGCTACGTGTACTACCGTGGT

GCGGGCGGCGGCATGGGCGCGGGGGCAGCGGCTGTAGCCTCGGCTGGGGT

CATCTATCCCTTCCAGCCCCGGGCGCGTTACGAGGACTACGGAGGTGGAG

GAGGCGAGGAGCAGCCCGAGTACCCGGCCCAGGGCTTTTACCCAGCCCCA

GAGAGGCCCTATGTGCCCCAGCCCCAACCCCAGCCCCAGCCCCAGCCGCA

GCCTCAGCCCCAACCTTCCGACGGCCTTGACCGTCGTTACTCGCACAGCC

TGTACAATGAAGGCACCCCGGGATTCGAGCAGGCCTACCCTGATCCCAGC

ACCGACGTGTCGCAGGCCCCCGCGGGGGCCGGTGGCACCTACGGTGGCGC

TGGCGATCCTCGCCTCGGCTGGTACCCGCCCTATGCAGCCAATGTGCCAC

CGGAGGCCTACGTCCCTCCGCGTGCGGTGGAGCCACAGCCCCCATTCCGC

GTGCTGGAGCCACCTTACTTGCCCGTGCGAAGCTCTGATGCGCCCTCCCA

GGGTGGGGAGCGGAACGGCGCCCAGCAGGGCCGTCTCAGCGTGGGTAGTG

TGTACCGACCCAACCAGAATGGTCGCGgtgagtacaatccacgagcgcct

tggctcgacattttggccactggtgaattgctggtgcggaccctgtttgt

ttcttatcaaaggccttccagtggtgg

***Loxl1^em2Andm^* (58-bp del)** = 58-bp coding sequence deletion in the targeted exon. Predicted to cause frameshift and premature STOP in the targeted exon.

>mm39_ncbiRefSeqCurated_NM_010729.3_0 range=chr9:58218869-58220595 5'pad=100 3'pad=100 strand=- repeatMasking=none

ggacaggccacatagctaaaacccggcgggccgtagggccccgcggagga

ggccccagcaggcggaccaggtttctgaaacctccagacactttcagcct

GTTGCTTATTCATTCAGAGTGGGAAAGCGCCAGCCTAGCGAGCAGCCAGC

CAGTGTGGGGCTGGCCATGTAAGGGCCCCAGGAGGTCCTGCCCGTCCCGT

GCCCTACGGAAAGCCTCGTGCAGCCCTGGGCACCGCCCCTGCCCTGCCCT

GACCCCTTGGCCTCGAAATGCTGTCATCGGAGGAGCCGTCCCGCTCGGGA

CAAGGCCAGGATGGACAAAGCTAGAGCTTGGGCAGGCGAAGAGCCATCCT

GTCCTCGAGGCCGTGGGAAGAGAAGCACGCACCGAGGCCCACTCTCTGCC

CACCCGGCCTCTGCAGAAGGGCCACTATGGCTCTGGCCGGAGCCGGCAGC

CAGCTGAGGACCCTGGTGTGGAGTGCCTGCCTGTGCGTCCTGGTGCACGG

GCAGCAAGCGCAGCCAGGACAGGGCTCGGACCCAGGACGCTGGCGGCAGC

TGATCCAGTGGGAGAACAACGGGCAGGTGTACAGCTTGCTCAACTCGGGC

TCTGAGTACGTGCCCGCGGGGCCCCAACGAGGGGAGACTAGCTCCAGGGT

TCTACTGGCAGGCGCGCCCCAAACGTCGCAGCGGCGCAGTCAGGGGGGAC

CCCGGCGTCGGCAGGCGCCGTCCCTGCCCCTGCCCGGACGCGTGGGTTCG

GACACTGTGCGCGGCCAAACGCGGCACCCGTTCGGCTTCGGCCAGGTGCC

CGACAACTGGAGAGAGGTGGCCGTCGGGGACAGTACCGGCATGGCTCGGG

CCCGCACCTCCGTCTCCCAGCAACGGCACGGGGGCTCCGCCTCCTCCTCG

GTTTCGGCCTCGGCCTTCGCCACCACCTATCGCCAGCCATCCTCCTACCC

GCAGCAGTTCCCCTATCCGCAGGCGCCCTTCGTAAACCAGTATGAGAACT

ACGATCCCGCGTCACGGACCTACGAACAGGGCTACGTGTACTACCGTGGT

GCGGGCGGCGGCATGGGCGCGGGGGCAGCGGCTGTAGCCTCGGCTGGGGT

CATCTATCCCTTCCAGCCCCGGGCGCGTTACGAGGACTACGGAGGTGGAG

GAGGCGAGGAGCAGCCCGAGTACCCGGCCCAGGGCTTTTACCCAGCCCCA

GAGAGGCCCTATGTGCCCCAGCCCCAACCCCAGCCCCAGCCCCAGCCGCA

GCCTCAGCCCCAACCTTCCGACGGCCTTGACCGTCGTTACTCGCACAGCC

TGTACAATGAAGGCACCCCGGGATTCGAGCAGGCCTACCCTGATCCCAGC

ACCGACGTGTCGCAGGCCCCCGCGGGGGCCGGTGGCACCTACGGTGGCGC

TGGCGATCCTCGCCTCGGCTGGTACCCGCCCTATGCAGCCAATGTGCCAC

CGGAGGCCTACGTCCCTCCGCGTGCGGTGGAGCCACAGCCCCCATTCCGC

GTGCTGGAGCCACCTTACTTGCCCGTGCGAAGCTCTGATGCGCCCTCCCA

GGGTGGGGAGCGGAACGGCGCCCAGCAGGGCCGTCTCAGCGTGGGTAGTG

TGTACCGACCCAACCAGAATGGTCGCGgtgagtacaatccacgagcgcct

tggctcgacattttggccactggtgaattgctggtgcggaccctgtttgt

ttcttatcaaaggccttccagtggtgg

***Loxl1^em3Andm^* (2-bp ins):** 2-bp coding sequence insertion in the targeted exon. Predicted to cause frameshift and premature STOP in the targeted exon.

>mm39_ncbiRefSeqCurated_NM_010729.3_0 range=chr9:58218869-58220595 5'pad=100 3'pad=100 strand=- repeatMasking=none

ggacaggccacatagctaaaacccggcgggccgtagggccccgcggagga

ggccccagcaggcggaccaggtttctgaaacctccagacactttcagcct

GTTGCTTATTCATTCAGAGTGGGAAAGCGCCAGCCTAGCGAGCAGCCAGC

CAGTGTGGGGCTGGCCATGTAAGGGCCCCAGGAGGTCCTGCCCGTCCCGT

GCCCTACGGAAAGCCTCGTGCAGCCCTGGGCACCGCCCCTGCCCTGCCCT

GACCCCTTGGCCTCGAAATGCTGTCATCGGAGGAGCCGTCCCGCTCGGGA

CAAGGCCAGGATGGACAAAGCTAGAGCTTGGGCAGGCGAAGAGCCATCCT

GTCCTCGAGGCCGTGGGAAGAGAAGCACGCACCGAGGCCCACTCTCTGCC

CACCCGGCCTCTGCAGAAGGGCCACTATGGCTCTGGCCGGAGCCGGCAGC

CAGCTGAGGACCCTGGTGTGGAGTGCCTGCCTGTGCGTCCTGGTGCACGG

GCAGCAAGCGCAGCCAGGACAGGGCTCGGACCCAGGACGCTGGCGGCAGC

TGATCCAGTGGGAGAACAACGGGCAGGTGTACAGCTTGCTCAACTCGGGC

TCTGAGTACGTGCCCGCGGGGCCCCAACGAGGGGAGACTAGCTCCAGGGT

TCTACTGGCAGGCGCGCCCCAAACGTCGCAGCGGCGCAGTCAGGGGGGAC

CCCGGCGTCGGCAGGCGCCGTCCCTGCCCCTGCCCGGACGCGTGGGTTCG

GACACTGTGCGCGGCCAAA **AA** CGCGGCACCCGTTCGGCTTCGGCCAGGTGCC

CGACAACTGGAGAGAGGTGGCCGTCGGGGACAGTACCGGCATGGCTCGGG

CCCGCACCTCCGTCTCCCAGCAACGGCACGGGGGCTCCGCCTCCTCCTCG

GTTTCGGCCTCGGCCTTCGCCACCACCTATCGCCAGCCATCCTCCTACCC

GCAGCAGTTCCCCTATCCGCAGGCGCCCTTCGTAAACCAGTATGAGAACT

ACGATCCCGCGTCACGGACCTACGAACAGGGCTACGTGTACTACCGTGGT

GCGGGCGGCGGCATGGGCGCGGGGGCAGCGGCTGTAGCCTCGGCTGGGGT

CATCTATCCCTTCCAGCCCCGGGCGCGTTACGAGGACTACGGAGGTGGAG

GAGGCGAGGAGCAGCCCGAGTACCCGGCCCAGGGCTTTTACCCAGCCCCA

GAGAGGCCCTATGTGCCCCAGCCCCAACCCCAGCCCCAGCCCCAGCCGCA

GCCTCAGCCCCAACCTTCCGACGGCCTTGACCGTCGTTACTCGCACAGCC

TGTACAATGAAGGCACCCCGGGATTCGAGCAGGCCTACCCTGATCCCAGC

ACCGACGTGTCGCAGGCCCCCGCGGGGGCCGGTGGCACCTACGGTGGCGC

TGGCGATCCTCGCCTCGGCTGGTACCCGCCCTATGCAGCCAATGTGCCAC

CGGAGGCCTACGTCCCTCCGCGTGCGGTGGAGCCACAGCCCCCATTCCGC

GTGCTGGAGCCACCTTACTTGCCCGTGCGAAGCTCTGATGCGCCCTCCCA

GGGTGGGGAGCGGAACGGCGCCCAGCAGGGCCGTCTCAGCGTGGGTAGTG

TGTACCGACCCAACCAGAATGGTCGCGgtgagtacaatccacgagcgcct

tggctcgacattttggccactggtgaattgctggtgcggaccctgtttgt

ttcttatcaaaggccttccagtggtgg

***Loxl1^em4Andm^* (27-bp del + 1-bp del):** 27-bp coding sequence deletion and a separate 1-bp coding sequence insertion, both in the targeted exon. Predicted to cause frameshift and premature STOP in the targeted exon.

>mm39_ncbiRefSeqCurated_NM_010729.3_0 range=chr9:58218869-58220595 5'pad=100 3'pad=100 strand=- repeatMasking=none

ggacaggccacatagctaaaacccggcgggccgtagggccccgcggagga

ggccccagcaggcggaccaggtttctgaaacctccagacactttcagcct

GTTGCTTATTCATTCAGAGTGGGAAAGCGCCAGCCTAGCGAGCAGCCAGC

CAGTGTGGGGCTGGCCATGTAAGGGCCCCAGGAGGTCCTGCCCGTCCCGT

GCCCTACGGAAAGCCTCGTGCAGCCCTGGGCACCGCCCCTGCCCTGCCCT

GACCCCTTGGCCTCGAAATGCTGTCATCGGAGGAGCCGTCCCGCTCGGGA

CAAGGCCAGGATGGACAAAGCTAGAGCTTGGGCAGGCGAAGAGCCATCCT

GTCCTCGAGGCCGTGGGAAGAGAAGCACGCACCGAGGCCCACTCTCTGCC

CACCCGGCCTCTGCAGAAGGGCCACTATGGCTCTGGCCGGAGCCGGCAGC

CAGCTGAGGACCCTGGTGTGGAGTGCCTGCCTGTGCGTCCTGGTGCACGG

GCAGCAAGCGCAGCCAGGACAGGGCTCGGACCCAGGACGCTGGCGGCAGC

TGATCCAGTGGGAGAACAACGGGCAGGTGTACAGCTTGCTCAACTCGGGC

TCTGAGTACGTGCCCGCGGGGCCCCAACGAGGGGAGACTAGCTCCAGGGT

TCTACTGGCAGGCGCGCCCCAAACGTCGCAGCGGCGCAGTCAGGGGGGAC

CCCGGCGTCGGCAGGCGCCGTCCCTGCCCCTGCCCGGACGCGTGGGTTCG

GACACTGTGCGCGGCCAAACGCGGCACCCGTTCGGCTTCGGCCAGGTGCC

CGACAACTGGAGAGAGGTGGCCGTCGGGGACAGTACCGGCATGGCTCGGG

CCCGCACCTCCGTCTCCCAGCAACGGCACGGGGGCTCCGCCTCCTCCTCG

GTTTCGGCCTCGGCCTTCGCCACCACCTATCGCCAGCCATCCTCCTACCC

GCAGCAGTTCCCCTATCCGCAGGCGCCCTTCGTAAACCAGTATGAGAACT

ACGATCCCGCGTCACGGACCTACGAACAGGGCTACGTGTACTACCGTGGT

GCGGGCGGCGGCATGGGCGCGGGGGCAGCGGCTGTAGCCTCGGCTGGGGT

CATCTATCCCTTCCAGCCCCGGGCGCGTTACGAGGACTACGGAGGTGGAG

GAGGCGAGGAGCAGCCCGAGTACCCGGCCCAGGGCTTTTACCCAGCCCCA

GAGAGGCCCTATGTGCCCCAGCCCCAACCCCAGCCCCAGCCCCAGCCGCA

GCCTCAGCCCCAACCTTCCGACGGCCTTGACCGTCGTTACTCGCACAGCC

TGTACAATGAAGGCACCCCGGGATTCGAGCAGGCCTACCCTGATCCCAGC

ACCGACGTGTCGCAGGCCCCCGCGGGGGCCGGTGGCACCTACGGTGGCGC

TGGCGATCCTCGCCTCGGCTGGTACCCGCCCTATGCAGCCAATGTGCCAC

CGGAGGCCTACGTCCCTCCGCGTGCGGTGGAGCCACAGCCCCCATTCCGC

GTGCTGGAGCCACCTTACTTGCCCGTGCGAAGCTCTGATGCGCCCTCCCA

GGGTGGGGAGCGGAACGGCGCCCAGCAGGGCCGTCTCAGCGTGGGTAGTG

TGTACCGACCCAACCAGAATGGTCGCGgtgagtacaatccacgagcgcct

tggctcgacattttggccactggtgaattgctggtgcggaccctgtttgt

ttcttatcaaaggccttccagtggtgg

***Loxl1^em5Andm^* (39-bp del + 8-bp del):** 39-bp coding sequence deletion and a separate 8-bp coding sequence insertion, both in the targeted exon. Predicted to cause frameshift and premature STOP in the targeted exon.

>mm39_ncbiRefSeqCurated_NM_010729.3_0 range=chr9:58218869-58220595 5'pad=100 3'pad=100 strand=- repeatMasking=none

ggacaggccacatagctaaaacccggcgggccgtagggccccgcggagga

ggccccagcaggcggaccaggtttctgaaacctccagacactttcagcct

GTTGCTTATTCATTCAGAGTGGGAAAGCGCCAGCCTAGCGAGCAGCCAGC

CAGTGTGGGGCTGGCCATGTAAGGGCCCCAGGAGGTCCTGCCCGTCCCGT

GCCCTACGGAAAGCCTCGTGCAGCCCTGGGCACCGCCCCTGCCCTGCCCT

GACCCCTTGGCCTCGAAATGCTGTCATCGGAGGAGCCGTCCCGCTCGGGA

CAAGGCCAGGATGGACAAAGCTAGAGCTTGGGCAGGCGAAGAGCCATCCT

GTCCTCGAGGCCGTGGGAAGAGAAGCACGCACCGAGGCCCACTCTCTGCC

CACCCGGCCTCTGCAGAAGGGCCACTATGGCTCTGGCCGGAGCCGGCAGC

CAGCTGAGGACCCTGGTGTGGAGTGCCTGCCTGTGCGTCCTGGTGCACGG

GCAGCAAGCGCAGCCAGGACAGGGCTCGGACCCAGGACGCTGGCGGCAGC

TGATCCAGTGGGAGAACAACGGGCAGGTGTACAGCTTGCTCAACTCGGGC

TCTGAGTACGTGCCCGCGGGGCCCCAACGAGGGGAGACTAGCTCCAGGGT

TCTACTGGCAGGCGCGCCCCAAACGTCGCAGCGGCGCAGTCAGGGGGGAC

CCCGGCGTCGGCAGGCGCCGTCCCTGCCCCTGCCCGGACGCGTGGGTTCG

GACACTGTGCGCGGCCAAACGCGGCACCCGTTCGGCTTCGGCCAGGTGCC

CGACAACTGGAGAGAGGTGGCCGTCGGGGACAGTACCGGCATGGCTCGGG

CCCGCACCTCCGTCTCCCAGCAACGGCACGGGGGCTCCGCCTCCTCCTCG

GTTTCGGCCTCGGCCTTCGCCACCACCTATCGCCAGCCATCCTCCTACCC

GCAGCAGTTCCCCTATCCGCAGGCGCCCTTCGTAAACCAGTATGAGAACT

ACGATCCCGCGTCACGGACCTACGAACAGGGCTACGTGTACTACCGTGGT

GCGGGCGGCGGCATGGGCGCGGGGGCAGCGGCTGTAGCCTCGGCTGGGGT

CATCTATCCCTTCCAGCCCCGGGCGCGTTACGAGGACTACGGAGGTGGAG

GAGGCGAGGAGCAGCCCGAGTACCCGGCCCAGGGCTTTTACCCAGCCCCA

GAGAGGCCCTATGTGCCCCAGCCCCAACCCCAGCCCCAGCCCCAGCCGCA

GCCTCAGCCCCAACCTTCCGACGGCCTTGACCGTCGTTACTCGCACAGCC

TGTACAATGAAGGCACCCCGGGATTCGAGCAGGCCTACCCTGATCCCAGC

ACCGACGTGTCGCAGGCCCCCGCGGGGGCCGGTGGCACCTACGGTGGCGC

TGGCGATCCTCGCCTCGGCTGGTACCCGCCCTATGCAGCCAATGTGCCAC

CGGAGGCCTACGTCCCTCCGCGTGCGGTGGAGCCACAGCCCCCATTCCGC

GTGCTGGAGCCACCTTACTTGCCCGTGCGAAGCTCTGATGCGCCCTCCCA

GGGTGGGGAGCGGAACGGCGCCCAGCAGGGCCGTCTCAGCGTGGGTAGTG

TGTACCGACCCAACCAGAATGGTCGCGgtgagtacaatccacgagcgcct

tggctcgacattttggccactggtgaattgctggtgcggaccctgtttgt

ttcttatcaaaggccttccagtggtgg

***Rbms3* (NM_001172121.1)**

Showing exon 3 and 100 bp of flanking introns

Yellow highlight = deleted

Capital case = exons

***Rbms3^em1Andm^* (13-bp del):** 13-bp deletion encompassing the splice acceptor and the first 8 coding bases of the targeted exon. Predicted to disrupt splicing.

>mm39_ncbiRefSeqCurated_NM_001172121.1_4 range=chr9:116938929-116939283 5'pad=100 3'pad=100 strand=- repeatMasking=none

caagcagcttacctttagtgaatgcttctgacctgtgactaggccctggc

ttctgcttgtccagtaacggtttgttttgtttttcttctgtttgcagcag

TCCTATGCACCAGCTCCCCACCCCATGGCTCCTCCCAGCCCCAGCACAAA

CAGCAGCAGCAACAGCAGCGGGGAACAGTTGAGTAAGACAAACCTATACA

TCCGAGGACTTCCACCAGGCACCACTGACCAGGACCTCATCAAGCTATGT

CAACCgtaagtgtcctgaggctccttgggcctgttcagatgcacatatca

cctgtggacaccctgaagggctgctgtacccactgcagactcaatgatga

agcct

***Sema6a* (NM_018744.2)**

Showing exon 1 and 100 bp of flanking introns

Red text = inserted

Yellow highlight = deleted

Capital case = exon

***Sema6a^em1Andm^* (1-bp ins):** 1-bp coding sequence insertion in the targeted exon. Predicted to cause frameshift and premature STOP in the next downstream exon.

>mm39_ncbiRefSeqCurated_NM_018744.2_4 range=chr18:47437071-47437388 5'pad=100 3'pad=100 strand=- repeatMasking=none

gccctcctcctcctcttcctcctcctcctcgaactggccccattgattct

tggcttgatctgcatttccctctcttaccccgtctcctcctttttcacag

ATACAAAACAGTATCCGGTGTTTGTGGGCCACAAGCC **A** AGGACGGAACACC

ACGCAGAGGCACAGGCTGGACATCCAGATGATCATGATCATGAACAGAAC

CCTCTACGTTGCTGCTCGgtaagagacattccttgcctgcaccagcagag

gacctgaaggttcctgtccccacccacccacggtgcatctttctaagaac

ttgaggtcccttactatg

***Sema6a^em2Andm^* (20-bp del):** 20-bp coding sequence deletion in the targeted exon. Predicted to cause frameshift and premature STOP in the next downstream exon.

>mm39_ncbiRefSeqCurated_NM_018744.2_4 range=chr18:47437071-47437388 5'pad=100 3'pad=100 strand=- repeatMasking=none

gccctcctcctcctcttcctcctcctcctcgaactggccccattgattct

tggcttgatctgcatttccctctcttaccccgtctcctcctttttcacag

ATACAAAACAGTATCCGGTGTTTGTGGGCCACAAGCCAGGACGGAACACC

ACGCAGAGGCACAGGCTGGACATCCAGATGATCATGATCATGAACAGAAC

CCTCTACGTTGCTGCTCGgtaagagacattccttgcctgcaccagcagag

gacctgaaggttcctgtccccacccacccacggtgcatctttctaagaac

ttgaggtcccttactatg

***Sema6a^em3Andm^* (12-bp del):** 12-bp coding sequence deletion in the targeted exon. Predicted to cause in-frame deletion of 4 amino acids in the targeted exon.

>mm39_ncbiRefSeqCurated_NM_018744.2_4 range=chr18:47437071-47437388 5'pad=100 3'pad=100 strand=- repeatMasking=none

gccctcctcctcctcttcctcctcctcctcgaactggccccattgattct

tggcttgatctgcatttccctctcttaccccgtctcctcctttttcacag

ATACAAAACAGTATCCGGTGTTTGTGGGCCACAAGCCAGGACGGAACACC

ACGCAGAGGCACAGGCTGGACATCCAGATGATCATGATCATGAACAGAAC

CCTCTACGTTGCTGCTCGgtaagagacattccttgcctgcaccagcagag

gacctgaaggttcctgtccccacccacccacggtgcatctttctaagaac

ttgaggtcccttactatg

***Tlcd5 (NM*_*001034863.3)***

Showing exon 2 and 100 bp of flanking introns

Red text = inserted

Yellow highlight = deleted

Capital case = exon

***Tlcd5^em1Andm^* (1-bp ins + 11-bp del):** 1-bp coding sequence insertion and a separate 11-bp coding sequence deletion in the targeted exon. Predicted to cause frameshift and premature stop in the targeted exon.

>mm39_ncbiRefSeqCurated_NM_001034863.3_2 range=chr9:43024656-43025055 5'pad=100 3'pad=100 strand=- repeatMasking=none

ttgttttatagtagcaactggggcatgtgcacaaaatgagcggcttctgg

tttcttggggtacattctttgttctcttggttttgttctctctgtcatag

GATGGCAGTAGGTCTCTGTGTGCAGGTGCTGTGCAGCCTGGGTGGCTGGC

TCTCACTCTATACATCTTTCTGCTGCCTGAACAAGCACCGAAGCTGTGAG

TGGAGCTGTC **A** GGCTGGTGACCTTCACCCACGGAGTCCTCTCCATAGGTCT

GTCTGCTTATATTGGCTTCATCGATGGCCCTTGGCCTTTTACCCACCCAG

gtaggtagaaggggcattagagatattttcctaagagttttatgatttag

gggcttgaaaaaagataaatttgtgttacattgaagaataattcccattc
