# Supplementary material for: Lack of evidence for GWAS signals of exfoliation glaucoma working via monogenic loss-of-function mutation in the nearest gene
Source: Hum Mol Genet. 2024 May 20;33(17):1481–94. doi: 10.1093/hmg/ddae088 (PMC13142156; doi:10.1093/hmg/ddae088)
Supplement: Supplemental_Figure_Legends_ddae088 [file supplemental_figure_legends_ddae088.docx]

**Supplemental File Legends**

**Supplemental File 1. Table of slit-lamp data (.xlsx)**

**Supplemental File 2. Representative images showing anterior segment slit-lamp images from young time point mice.** Empty fields indicate no data for a particular combination. Note that no indices of XFS were present and eyes were typically healthy in appearance. The *Cacna1a* homozygote has a smaller eye but is also younger than other mice in the panel (24 days). All mice = ~1–2 month. Alt text: Collection of multiple ophthalmic image types collected from *Loxl1* mutant mice showing phenotypes of the anterior eye, posterior eye, and conjunctiva.

**Supplemental File 3. Table of individual SD-OCT data (.xlsx)**

**Supplemental File 4. Table of group SD-OCT data (.xlsx)**

**Supplemental File 5. Representative anterior segment SD-OCT images from early time point mice.** Empty fields indicate no data for a particular genotype. Note that gross qualitative appearances are similar across genotypes. The *Cacna1a* homozygote has a notably shallow anterior chamber depth but is also younger than other mice in the panel (24 days). There is a recurrent modest increase in anterior chamber depth for *Loxl1* homozygotes, which is particularly evident in panel O. All mice = ~1–2 month. Alt text: Figure with a collection of optical coherence tomography images from 1 to 2 month old mice showing anterior segment appearance for each iteration of gene, allele, and genotype of the study.

**Supplemental File 6. Representative retinal SD-OCT images from early point mice.** Empty fields indicate no data for a particular genotype. Note that gross qualitative appearances are similar across genotypes. A recurrent modest change in appearance of the ganglion cell complex is apparent in *Sema6a^em2Andm^* (G’) and *Sema6a^em1Andm^* (J’). All mice = ~1–2 month. Alt text: Figure with a collection of optical coherence tomography images from 1 to 2 month old mice showing retinal appearance for each iteration of gene, allele, and genotype of the study.

**Supplemental File 7. Sporadic phenotypes that were not correlated to specific genotypes. (A, B)** Microphthalmia, shown in paired images from the same eye. **(C, D)** Peters anomaly, shown in paired images from the same eye. **(E, F).** Pupil loop, shown in paired images from the same eye. Note, to help show this small feature, the slit-lamp image in panel **E** was collected at 40X magnification with a narrowed slit beam and the image had brightness enhanced in post-processing. **(G, H)** Avulsed retinal vessels. **(I)** Band keratopathy. **(J)** Retinal dysplasia. **(K)** Iris cyst. **(L)** Discontinuous corneal endothelium. **(M)** Iridocorneal adhesion concurrent with **(N)** retinal/optic nerve dysplasia. **(O, P)** Anterior vitreous opacity, shown in paired images from the same eye. **(Q, R)** Vitreous pigment clumps. **(S)** Retinal spotting. **(T)** Geographic atrophy. Images collected by slit-lamp **(A, C, E)**, anterior segment SD-OCT **(B, D, F, I, K, L, M)**, retinal SD-OCT **(H, J, N, O, Q)**, and fundus **(P, R, S, T)**. Alt text: Collection of multiple ophthalmic image types collected from various mice of the study showing examples of unusual sporadic phenotypes observed in some eyes.

**Supplemental File 8. Targeting strategies for candidates (.pdf).** Each map includes the reference RefSeq, a locus map based on the UCSC Genome Browser database annotations, and locations of the gRNAs used for targeting.

**Supplemental File 9. Detailed descriptions of mutations (.pdf).** Sequences shown from UCSC Genome Browser (GRCm39/mm39). Based on UCSC Annotation of RefSeq. Showing either the affected exon and 100 bp of flanking intronic sequences.

**Supplemental File 10. Table of cohorts (.xlsx).**
